# A scoping review on the use of natural language processing in research on political polarization: trends and research prospects

**DOI:** 10.1007/s42001-022-00196-2

**Published:** 2022-12-19

**Authors:** Renáta Németh

**Affiliations:** Research Center for Computational Social Science, Faculty of Social Sciences, ELTE Eötvös Loránd University, Budapest, Hungary

**Keywords:** Language polarization, Political polarization, Partisan language, Natural language processing, Text mining, Computational text analysis

## Abstract

As part of the “text-as-data” movement, Natural Language Processing (NLP) provides a computational way to examine political polarization. We conducted a methodological scoping review of studies published since 2010 (*n* = 154) to clarify how NLP research has conceptualized and measured political polarization, and to characterize the degree of integration of the two different research paradigms that meet in this research area. We identified biases toward US context (59%), Twitter data (43%) and machine learning approach (33%). Research covers different layers of the political public sphere (politicians, experts, media, or the lay public), however, very few studies involved more than one layer. Results indicate that only a few studies made use of domain knowledge and a high proportion of the studies were not interdisciplinary. Those studies that made efforts to interpret the results demonstrated that the characteristics of political texts depend not only on the political position of their authors, but also on other often-overlooked factors. Ignoring these factors may lead to overly optimistic performance measures. Also, spurious results may be obtained when causal relations are inferred from textual data. Our paper provides arguments for the integration of explanatory and predictive modeling paradigms, and for a more interdisciplinary approach to polarization research.

**Supplementary Information:**

The online version contains supplementary material available at 10.1007/s42001-022-00196-2.

## Introduction

### Language polarization – conceptualization and operationalization

As textual data sources grow in number and size, Natural Language Processing (NLP) is gaining ground in many social science subfields, including research on political polarization. In contrast to votes or polls, texts allow their authors to express a more nuanced opinion. Internet textual data reflect observed behavior as opposed to polls, and computational methods provide access to these vast amounts of data.

To review the most diverse approaches of polarization research, we wanted to refer to political polarizations in a broader sense. Indeed, characteristically different definitions of polarization can be found in the literature. Even one of the classic papers [1] treats polarization as multidimensional in character. According to the authors, polarization can be measured as (1) the dispersion of opinions, (2) the bi-modality of opinions, (3) the close association between different social attitudes, or (4) the correlation between social attitudes and salient individual characteristics. Lelkes [2] distinguishes two other forms: perceived polarization and affective polarization. A recent writing on the polarization of the digital sphere, Yarchi et al. [3], in addition to definitions already mentioned, distinguish interactional polarization that focuses on whether homophilic interactions are dominant over heterophilic ones (exploiting the network nature of the digital sphere). We included polarization not only around political ideologies, but also around public policy issues.

When searching for relevant studies, we also included “partisanship” as an alternative to "political polarization” in the search terms. Originally, partisanship was understood as affective or rational party identification [4], hence it could be causally linked to polarization, although it was not considered to be equivalent to it. Recently, however, many authors have been using partisanship and polarization as interchangeable concepts (e.g., [5]).

Turning to language polarization: Fiorina and Adams’ [6] comprehensive paper is one of the seminal works on political polarization, with over 1000 citations, listing different kinds of empirical evidence used to study political polarization, but not listing linguistic features. Linguistic manifestations of political polarization (*language polarization* for short from now on) has entered the scientific discourse at a later point, mostly in the last decade. The term “political polarization” has been first used at the largest conference of computational linguists (Annual Meetings of the Association for Computational Linguistics) in 2012.

When measuring language polarization, one either tries to adapt existing measurement practices of political polarization to textual data, or they develop a new approach. The former solution is possible for several traditionally used polarization measures. For example, DiMaggio et al.’s [1] measures, that are based on the distribution of a numerical variable, can be adapted if the textual data can be converted into numerical data in some appropriate way. As we will see in the review, affective polarization [2] and interactional polarization [3] can be also defined on textual data. In other cases, it is not possible to directly match the approach applied to texts with that applied to non-textual data, see e.g., topic choice, which, as the review shows, is often the focus of NLP analysis.

As the review will show, NLP methods most often introduce new ways for measuring polarization. The approaches differ according to the underlying conceptualization of political position and the data available. A focus of the review is on investigating whether researchers are indeed identifying traces of political polarization when they detect differences in language use between ideological sides—a question that arises in supervised classification.

### The aims of our paper

We provide a methodological scoping review on how researchers have used NLP to study language polarization. We identify data sources and computational techniques, and review the different conceptualizations and operationalizations of polarization.

Our review captures several features that describes the difference between the research paradigms of social science and computer science-based NLP. These two approaches can be best identified as explanatory and predictive in nature [7, 8]. Whether the research is embedded in theory, whether qualitative approach is also used, whether the study address causality, or whether the results are interpreted, all can be linked to this duality. Social scientists traditionally prioritize explanations, invoking causal mechanisms derived from theory. However, computer scientists are more concerned with developing accurate predictive models, leaving interpretability aside. The degree of integration of the two approaches is also characterized in the review.

The overarching aim of this scoping review is to provide a starting point for future research by synthesizing approaches, potential flaws, and solutions. We would also like to give social science researchers who are unfamiliar with NLP a picture of the ongoing research.

## The review’s methodology

We decided to write a scoping review because of the multidisciplinary nature of the papers to be reviewed, and because of the differences in terminology across disciplines. We performed the searches using Google Scholar, and included studies published between January 1, 2010 and June 29, 2021. Our initial search terms were political “polariz(s)ation” AND “natural language processing”, then added synonyms to both terms (for the detailed methodology of the review see the Supplement).

As is usually the case with scoping reviews, because of the undefined nature of the search terms, and because of searching anywhere within the article, we got many irrelevant hits, so a range of hard and soft exclusion criteria was defined. We synthesized our findings in a narrative report. Due to space limitations, technical details have been moved to the Supplementary Material.

## Results

### Summary

After deleting duplicates, our search returned 3078 unique hits, to which we added 6 more papers manually. Of the initial 3084 records, we identified 154 relevant studies (see Table S1 in the Supplement).

**Fig. 1:**
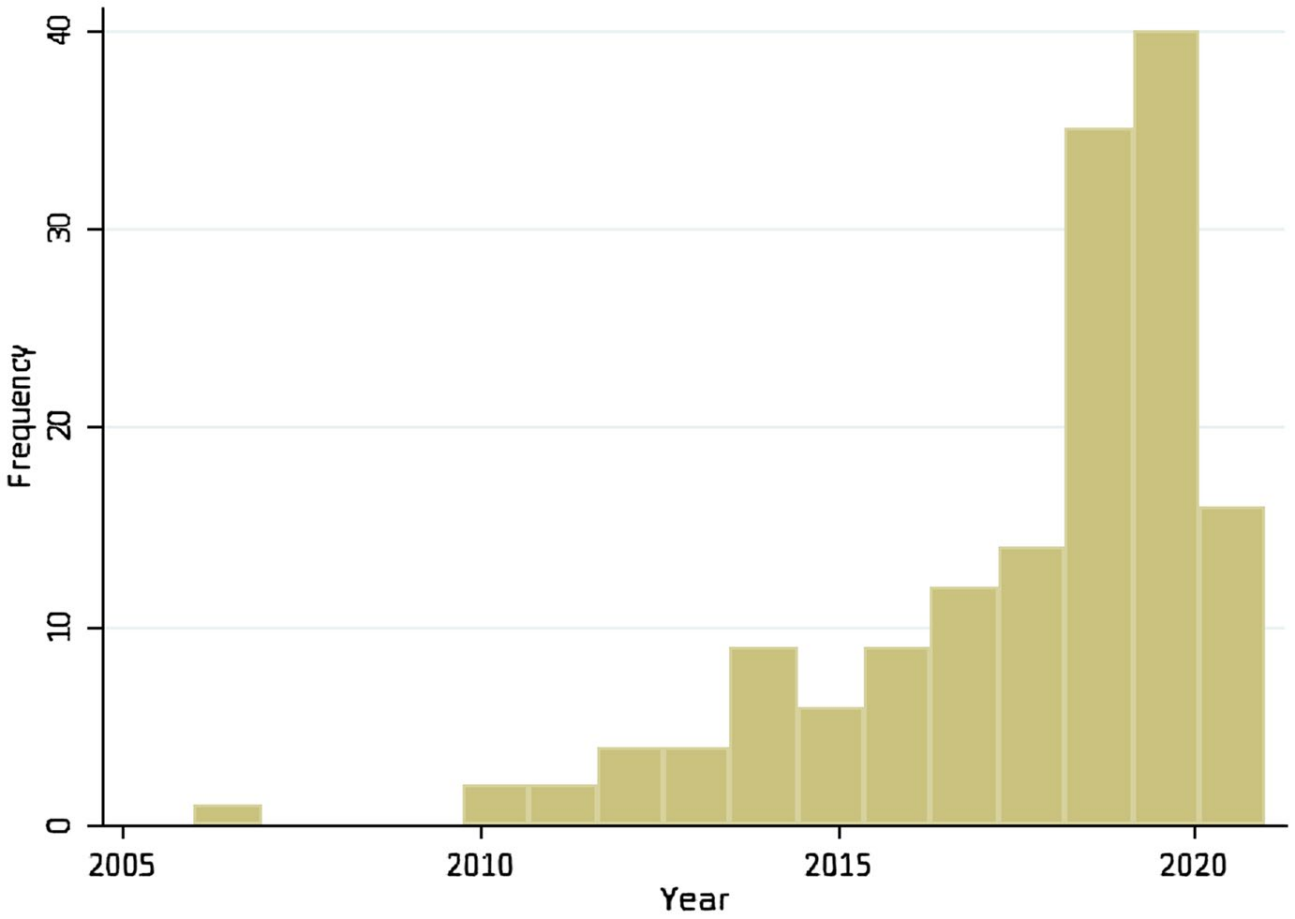
The included studies by year of publication

According to Fig. 1, the number of publications has risen during the past decade. Table 1 presents top 10 countries of the paper’s focus, and countries the authors are affiliated with. (Full information can be found in the Supplement, Table S2.) As Table 1 presents, more than half of the studies were conducted using US data and the US is also the most publishing country affiliated with half of the studies. The US dominance is probably a phenomenon that manifests itself throughout the scientific literature, and most explicitly in the areas with a strong methodological basis. Similar results suggesting US dominance were found by recent bibliometric analysis on carbon emissions from transport sector [9], on effects of COVID-19 pandemic on mental health [10], or in a more technical area, on NLP in medicine [11], finding US dominance of 29%, 21% and 63%, respectively.

**Table 1:** Top countries of the paper’s focus, and top countries the authors are affiliated with (with frequency of occurrence)

Country of paper’s focus		Affiliation country	
USA	91	USA	81
United Kingdom	9	Italy	14
Italy	6	United Kingdom	13
Spain	6	Germany	11
Canada	5	Qatar	10
Germany	5	Canada	8
India	3	Spain	7
Scotland	3	India	6
Turkey	3	Ireland	5
Ukraine	3	France	4

**Fig. 2:**
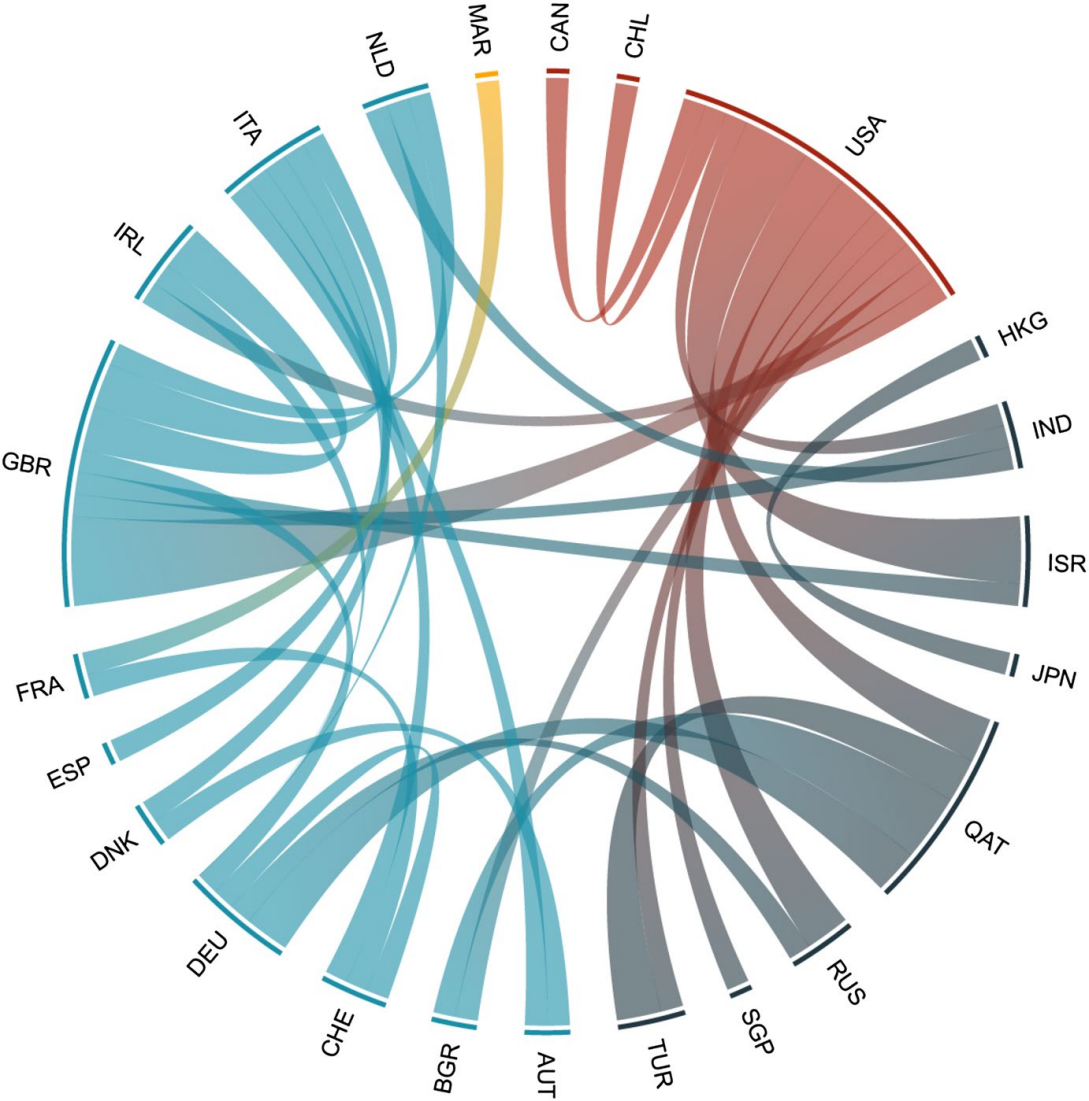
Co-authorship network of countries

Only a tenth of the studies were single-authored. Figure 2 demonstrates the research collaboration among countries (full information can be found in Table S4 of the Supplement). The network shows co-authorship relations. The size of a node indicates the number of studies affiliated to the country, the width of the edges is proportional to the number of publications produced in collaboration, and colors represent continents. Countries have been indicated by their corresponding three-letter abbreviation according to the ISO 3166 standard.

Figure 2 shows that in absolute numbers, the US leads in collaborations, GBR is second, and overall European and Asian countries are also very active. However, in terms of proportions (Table S4), it can be seen that 80% of US studies had no authors from other countries, while almost all of the non-US authors are collaborators, typically with a US co-author. These findings underline the US-centricity of the research topic.

**Fig. 3:**
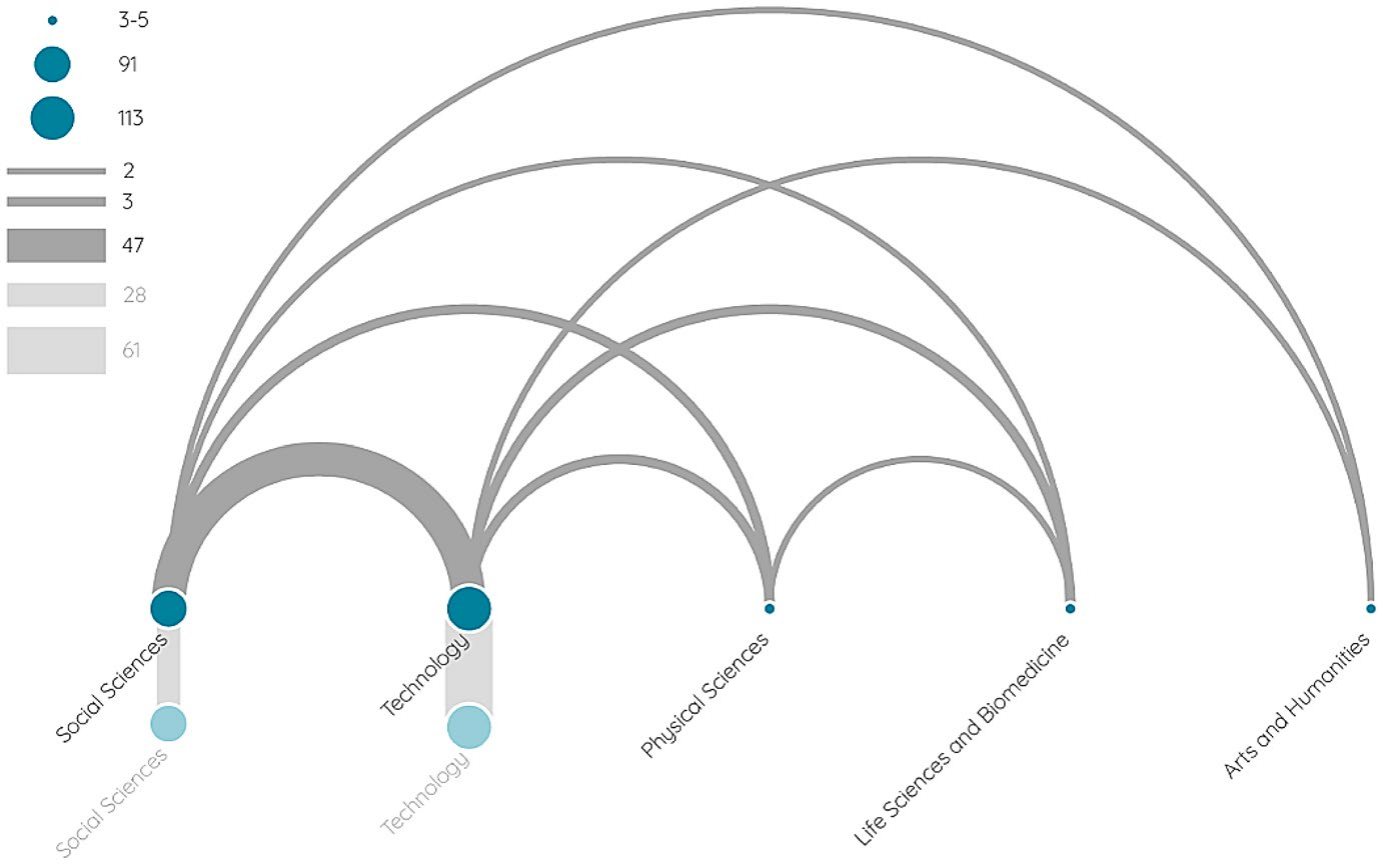
Co-authorship network of disciplines

Figure 3 represents co-authorship network of disciplines, its edges and nodes are defined in a similar way to Fig. 3, with disciplines instead of countries. Vertical edges at the bottom of the figure correspond to collaborations between authors from the same disciplines. (Table S4 and S5 in the Supplementary Material present full information and also describe the science classification method).

Two-thirds of the studies were assigned to Social Sciences and the same proportion to Technology. The number of non-interdisciplinary studies is very high: about one-fifth of the articles were written by social scientists only, and a further 40% (!) were written only with collaborators from the field of Technology. By merging Physical Sciences and Life Sciences & Medicine with Technology, we can conclude that 45% of the articles were co-authored exclusively from these areas, without subject matter collaborator.

As Table S1 shows, the range of sources is very diverse, with four fifths of the papers appearing in a source that is listed only once in the database, which indicates the multidisciplinary nature of the topic.

**Fig. 4:**
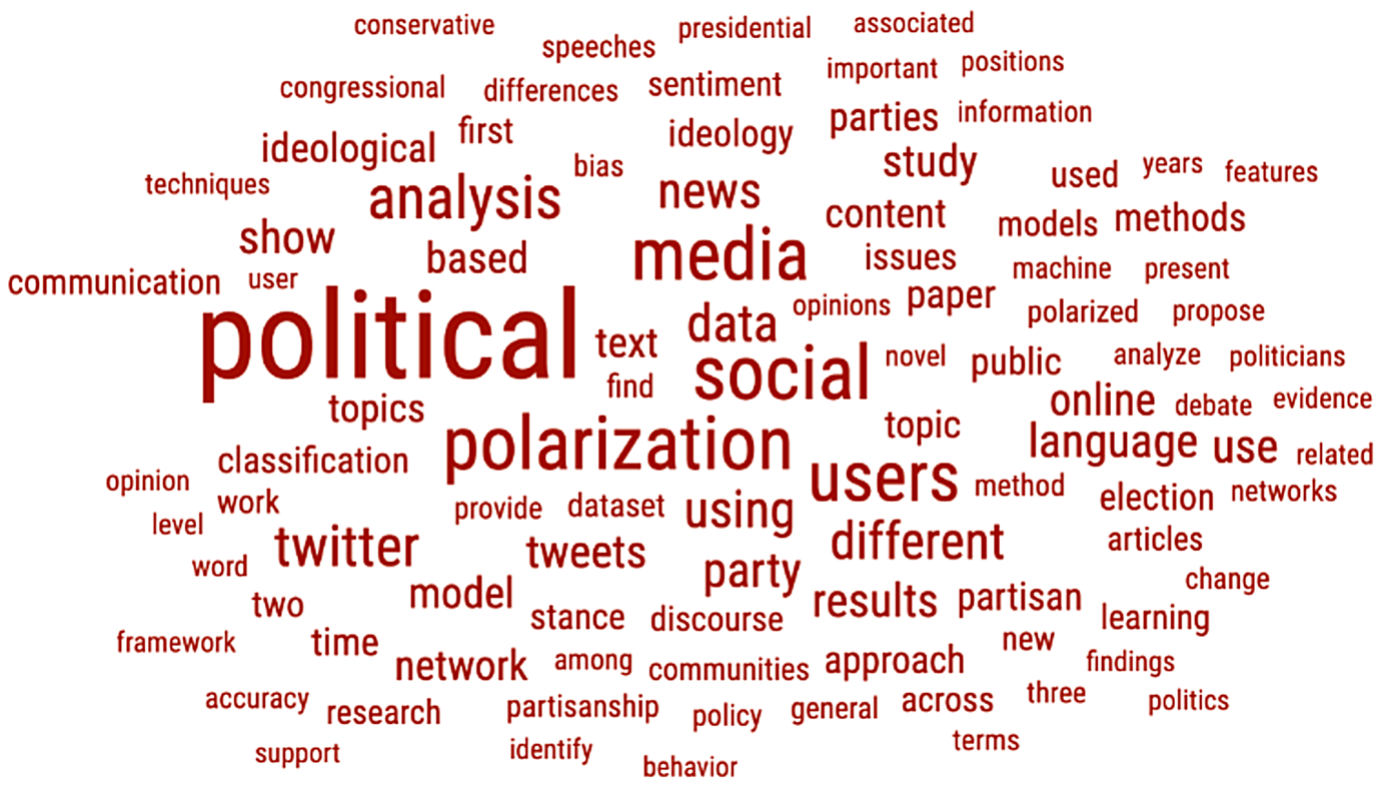
Word cloud of the studies’ abstract

Figure 4 presents a word cloud of the studies’ abstracts, the figure clearly shows the research focus.

### Data

#### Data sources

This section describes the data sources that the studies used. Choice of data source has an impact on the end result, we therefore registered the type of data sources (see Table 2 for the top 10, the full list is in the Supplement, Table S3.). According to Table 2, more than 40% of the studies used Twitter data. We agree with a reviewer's comment that when interpreting this result, different aspects of data usability should be taken into account. Twitter is the most accessible source of politically relevant texts in most countries. It is also much less time-consuming and requires less technical expertise to use than other sources. However, in addition to accessibility, the fruitfulness of the data should also be taken account—there are research questions for which the use of other sources, such as parliamentary speeches, may be much more justified.

**Table 2:** Top 10 data source, layers of political public sphere examined and length of period covered in the studies (with frequencies)

Type of data source		Which layers of the political public sphere are examined		Length of period covered	
Twitter	66	Lay only	63	Present data or period shorter than 2 years	105
Congressional/Parliament speeches	32	Official (politicians) only	50	2–5 years	9
News sites	22	Media only	19	5–10 years	11
Speeches outside Congress*	8	Expert only	5	10–20 years	8
Written public political documents**	7	Media and lay	4	20–50 years	10
Facebook	6	Official and media	4	50–100 years	2
Reddit	5	Official and lay	3	100 + years	9
Blogs	4	Official and expert	2		
Texts produced by non-political experts***	4	Official and media and expert	2		
Newspapers	3	Official and media and lay	1		

*Presidential/presidential candidates’ speeches, campaign speeches, public statements, presidential candidacy announcements, **party manifestos, candidate manifestos, party platforms, press releases, coalition agreements, ***judges’ written opinions, business reports, scientific papers)

Data sources also differ in the time period they cover, with longer time periods allowing for changes to be detected (see Table 2). Longer time scales also provide a basis for benchmarking the degree of polarization detected in the present. For example, Jensen et al. [12] found that although the political discourse became more polarized in the late 1990s, polarization remained low relative to the late 19th, and much of the twentieth century. Tracing changes over time is also possible by measuring changes in polarization not over a ‘common’ historical time span, but over the course of individual lives. For example, Iliev et al. [13] examined the legislative rhetoric of US Senators as a function of their time in office.

#### Layers of political discourse

Language polarization can appear at *different layers of the political public sphere*, including the official channels of political communication (e.g., parliamentary speeches); the different types of media, and also the user-generated contents (e.g., social media,). In the following, we will make a distinction between professional (official sources and the press) and non-professional (lay) communication. A fourth layer emerged during our reviewing process: the expert layer (e.g., texts written by judges). In this domain, ideological expressions are considered inappropriate, so it is a particularly intriguing question whether ideology can be detected by computational tools.

According to Table 2, most of the papers were concerned with the lay public (*n* = 71); they all used only social media data. The majority of them researched tweets or posts; exceptions were KhudaBukhsh et al. [14], who studied the comments sections of YouTube channels, or Wu et al. [15], who analyzed Twitter bios.

Studies that were concerned with the official layer (*n* = 60) were mostly based on legislative speeches. Exceptions were, for example, Gross and Jankowski [16], who used a dataset of local party manifestos in Germany, or Wang and Tucker [17], whose dataset consisted of press releases issued by members of the US Congress.

The expert sphere was studied by a smaller number of studies (*n* = 9). Among them were Jelveh et al. [18], who detected latent ideological bias in academic papers in economics, Diaf et al. [19], whose dataset consisted of business cycle report sections issued by German economic research institutes, and Hausladen et al. [20], who studied the ideological direction of US Circuit Court decisions.

Studies very rarely involved more than one layer of the public sphere (only 9%). Serrano-Contreras et al. [21] studied comments made on YouTube videos uploaded by politicians. This approach could be generally used to examine the reactions of one layer to another. Karamshuk et al. [22] studied both the media and the lay public during the Ukrainian–Russian conflict of 2013–14, but the two layers were researched separately.

Similarities between the polarization of different layers (e.g., whether partisan terms diffuse from one to the other) was rarely investigated. An exception was Hofmann et al. [23], who measured differences between the language of the political parties and their media representation. Acree [24], going even further, compared the structure of the layers he studied and showed that ideology represented in the expert discourse is rich and varied, but professional political debate compresses ideological expression into a single (left–right) dimension. Yan et al. [25, 26] studied three different layers, the expert sphere (conservative and liberal wikis), alongside with the media and politicians. Their main research question was to what extent the polarization of the three layers differed [25] and how well the models can be transferred across domains [26]. In the latter, they showed that models based on the Congressional Record have some success in classifying articles from the media, that is, there is a diffusion process between the Congress and the media.

#### Key lessons learned from data

Some papers call attention to an often-overlooked problem: the importance of *the nature of the data*. The way the data were collected and filtered, the context in which they were created, and the genre of the texts are all important factors that determine, for example, the performance of the model, or whether a machine learning model can be transferred from one database to another (a problem called transferability, or cross-domain generalizability).

Cohen and Ruths [27] proved that using politically discriminative hashtags to define the corpus (which is a standard research practice) favors including an artificially enriched population of politically polarized users, who can therefore be more easily classified by the model. Their result also suggests that previously reported performances had been systematically overoptimistic. They also showed that classifiers cannot be used to classify users outside the range of political orientation on which they were trained.

In other words, *groups of people with different political activities* also use very different languages. Therefore the common implicit assumption according to which the most extreme cases exhibit the same phenomenon, only in a more detectable way, does not stand. E.g., Diermeier et al. [28], Morini et al. [29], and Grover et al. [30] follow this assumption implicitly when selecting more explicit/extreme cases, as well as Cotelo et al. [31], who only included in their analysis clearly codable tweets that were given the same political label by the coders.

Yan et al. [25] achieved a result similar to that of Cohen and Ruths [27] on three text corpora of *different genres from different public spheres*. They studied different layers of political discourse, and found that although the models perform well on within-dataset, their ability to generalize from one dataset to another is poor. Another of their important results show that the success of prediction is not only domain-specific but also *time-specific*: effectiveness of prediction decreases as the test data is further removed in time from the training data. This raises questions about Diermeier et al.’s [28] methodology, whose training set consists of speeches in the 101st-107th Senates, and the test set comes from the 108th Senate.

Potthast et al. [32] made the important finding that the language of news sites is in fact not so much divided along right and left ideological lines, but rather along the *mainstream/hyper-partisan* dichotomy, i.e., along *stylistic lines*. Hirst et al. [33, 34] also detected poor transferability when classifying across two Canadian Parliaments (with the Liberal Party governing in the first case, and the Conservative Party in the second). It is partly this result that led them to the realization that *party status (being in opposition vs. being government)* is an often-overlooked factor when classifying texts by ideology.

### Methods

#### Text analytic methods

For the benefit of readers who are not familiar with text analytic methods, in the Supplement, we give a brief intuitive outline of the methods most used in the studies. Some of them are specific to political science (Wordfish, Wordscores, Wordshoal), others are general, widely used NLP methods (topic model, word embedding, supervised machine learning, sentiment analysis).

#### Operationalization of polarization

When operationalizing polarization, measurement of political positions must first be determined, then, based on it, measurement of polarization must be defined. Typically, one of the following two approaches were used to define the political position of a given text: ideological scaling or classification.

*Scaling* was mostly done using standard political science approaches (Wordscores, Wordfish, or Wordshoal, *n* = 8). Gross and Jankowski [16], using Wordfish on a dataset of local party manifestos in Germany, identified dimensions of party conflict. Medzihorsky et al. [35], using Wordfish, were able to show that the 2012 US Republican candidates moved farther away from the more traditional Republican ideology. Wordshoal was used, for example, by Goet [36].

Another approach to scaling comes from ideal point models (the DW-NOMINATE, [37], also belongs here). The models estimate political positions for legislators from legislative votes. The purpose of generalizing these models is that they can also incorporate texts. Nguyen et al. [38] introduced the hierarchical ideal point topic models, which uses not only votes but also associated bill text and the language of the legislators themselves and incorporates topic of the bills. Gerrish and Blei [39] develop the issue-adjusted ideal point model, which accounts for the contents of the bills. The idea is that the votes on a bill depend on a legislator’s general position, adjusted for the bill’s content.

When using scaling, degree of polarization can be approximated from the distribution of positions. In this context, an explicit measure is defined by Goet [36], which captures the consistency with which MPs fall within their party label across multiple policy issues. A score of “1” represents perfect polarization, with zero overlap between left and right wing parties.

Scaling has been used more by political science authors, while others used *classification* (51 studies used classification, mostly a supervised version). Classification in this context is a method that tries to identify the position of an author based on the words he or she has used. These studies, explicitly or implicitly, consider polarization as a classification problem. High classification performance suggests that language used on one political side is homogeneous, and different from language used on other sides. A polarization metrics can be defined: the greater the classification model’s ability to identify the position of the author, the greater polarization there is. Works written most explicitly in this approach are Goet [36] and Green et al. [40]. Following this approach, Bayram et al. [41] analyzed House of Representatives floor speeches, and detected a clear upward trend in classification performance, indicating that polarization have become more obvious in the language of the speeches. In their highly cited paper, Gentzkow et al. [42], building on methods developed by Taddy [43, 44], recommend a polarization measure that follows a similar logic. They specify a multinomial model of speech with choice probabilities that vary by party, and polarization is measured by how easily an observer who knows the model can guess a speaker's party just from the speaker’s choice of a single phrase. Kelly et al. [45] developed this method further.

In addition to scaling and supervised classification, other less common methods were also used. Samantray and Pin [46] used the ideological divergence indicator developed by Lelkes [2] that characterizes the level of polarization based on bi-modality of the distribution of a numerical variable. The latter variable traditionally comes from opinion polls, here it is generated as a feature of texts. Darwish [47] used a polarization measure on Twitter data following Garimella et al. [48], measuring the amount of controversy from characteristics of the conversation graph. Budhiraja and Pal [49] represented each politician as a word embedding vector based on the content of their tweets, then identified polarization as the partitioning of the resulting point cloud by party. Villa-Cox et al. [50], generalizing KhudaBukhsh et al. [14], interpreted polarization through machine translation. Their framework assumes that two sub-communities are speaking in two different languages and obtains single-word translations. Number of disagreed word-pairs present a quantifiable measure of polarization. Other proposals have been also made to operationalize polarization, see Gross et al. [51], and Acree et al. [52].

#### Methods used for examining changes over time

Most studies investigating changes over time simply divide the time interval into several sections, and carry out the same analysis on each section separately. Other studies combined NLP with traditional time modeling statistical methods. For example, Tsur et al. [53] applied time series regression on topic affiliations resulting from the topic model. Gross and Jankowski [16] used linear mixed-effects models with the dependent variable being the positions estimated by Wordfish. Hofmann et al. [23] employed time series modeling using generalized additive models to compare the lexical differences between parties.

There were studies using the structural topic model (STM), an NLP model that directly incorporates time. Farrell et al. [54], for example, analyzed texts written by US organizations about climate change over a 20-year period, and applied STM to examine how corporate funding ties influenced the change in topics over time.

Other authors, for example Gross et al. [51], Acree et al. [52], and Iliev et al. [13] developed their own statistical models to identify temporal changes in ideological positions.

#### Classification models

##### Unsupervised classification

The main advantage of unsupervised classification is that it does not require any prior labeling of users, therefore there is no need for domain knowledge. Stefanov et al. [55] and Darwish et al. [56] used unsupervised user stance detection on Twitter. After projecting users onto a low-dimensional space, they applied clustering, which allowed them to find core users who were representative of the different stances. Another example for unsupervised classification is cluster analysis, applied for example by Giglietto et al. [57].

##### Supervised classification

We have reviewed the relevant studies according to several criteria: nature of the target variable, way of annotation, use of structural information, and nature of classification features.

According to different definitions of political position, different target variables were used: stance [58], party [15], or ideology [59]. Specific target variable was used, for example, by Gerrish and Blei [39], who predicted the vote cast based on a legal text. The paper is unique in that it predicts the reaction to a text, not the author’s ideological position. Cotelo et al. [31] used stance on two parties instead of using it on only one (with positive, negative or neutral stances on each) as target variables, and defined the task as classifying tweets into any of the nine combinatorial categories.

The creation of labels for supervised learning (or “annotation”) is also an important issue, as obtaining labeled texts can itself be challenging. A *manually labeled* corpus to predict the ideological direction of US circuit court decisions was used by Hausladen et al. [20], with annotation being obviously a challenge in this case. If the annotation task is easy to teach, *crowdsourcing* can be used, like in the case of Wang and Tucker [17], who used Amazon’s Mechanical Turk.

In many other cases, manual annotation is not necessary, because the labels can be obtained from external data. This is obviously the case with politicians' texts. A less trivial example was given by Jelveh et al. [18], who investigated academic articles written by American economists, and determined the author's political leaning based on their political campaign contributions and petition signing activities. Zubiaga et al. [60] classified the stance of Twitter users on the independence movement in Catalonia, and their labels relied on users’ self-reported territory that they claim to be citizens of, which is directly indicative of their stance toward the independence movement.

In other cases, the label is inherited from larger units of analysis by smaller ones. Kulkarni et al. [61] used the classification of news sources provided by AllSides.com (an American company that assesses the political leaning of prominent media outlets), and applied labels to articles according to their sources. Karamshuk et al. [22] applied manual labeling on news sources, and labeled Twitter users based on the news sources they shared. Rao et al. [62] continued the chain of inheritance even further, carrying over the labels from web domains to users, and then from users to other users based on retweet patterns. However, Kobayashi et al. [63] pointed out that the above inheritance-based solutions assume that Twitter users prefer to follow media and politicians whose ideological positions are similar to their own, and these assumptions are not necessarily true.

In addition to the texts, some papers also exploited the *structural information* on the database for classification. This is especially feasible for social media data, where a network of relationships between users is available. It had been commonly found that involving structural information increases classification accuracy [31, 64]. Wang et al. [65] classified tweets and found that the best model is one that integrates texts and *pictures*. However, it is worth noting that if the primary goal is to study language polarization (instead of defining the “best” classifier), the importance of language itself should be examined. What may be a relevant question, however, is the fraction of linguistic information that constitutes total polarization.

Another relevant question is *what features were used*, if text-based classification was applied. Most often a bag-of-words approach or n-grams were used, ignoring syntax [41, 52]. Newer models model the compositional aspect of language, e.g., by applying a neural network framework [61, 66].

Several papers used pre-defined text properties as features in addition to the raw text. Potthast et al. [32] conducted a study of hyper-partisan news, and employed different stylometric features, such as readability scores, ratios of quoted words, number of paragraphs, etc. Hashtags proved to improve classifiers’ performance in several studies [64]. As hashtags are brief and information rich, they may reduce noise.

Word embedding was used for creating features in some studies [22, 60, 62, 65]. Zubiaga et al. [60] used word embedding for dimension reduction: word embedding representation of the content of a user’s timeline was used as a feature. Karamshuk et al. [22] illustrate another important application of word embedding: their starting dictionary consisted of terms considered to be indicators of partisan rhetoric, and these words were then matched to the most similar ones according to their word embedding representation to get the final feature set.

Other studies used the output of a topic model [58, 62], author-level text features [27], or media-level features [67]. Baly et al. [67] illustrate how to utilize texts from a variety of different sources: the authors’ aim was to classify the political ideology of news articles, and they determined media-level features based on the word embedding representation of (1) the bios of the medium’s Twitter followers, and (2) the content of the Wikipedia page describing the medium, and (3) Web-traffic information about the medium’s website.

#### Topic modeling

One in six studies applied topic modeling. The main topics and the words typical of the topics were often used for frame analysis [68]. According to Tsur et al. [53], topic co-occurrence can also approximate the way topics are framed. In other cases, see e.g., Sinno et al. [69], topic modeling was used only for technical reason to create a topically coherent corpus by omitting articles that were not related to the relevant topics.

Structural topic models are advantageous because they allow the incorporation of document metadata that affects how the topics vary by document. Including financial metadata allowed Farrell et al. [54] to test the effects of corporate funding on how organizations discuss climate change.

Others proposed the introduction of new types of topic models, e.g., Thonet et al. [70], Trabelsi and Zaiane [71], and Koylu et al. [72].

#### Sentiment analysis

One in six studies applied sentiment analysis. Most often *dictionary-based sentiment analysis* was used [20, 73, 74]. Grover et al. [30] for example examined moral, affective, and cognitive differences in language use between the two opposing sides of the debate over immigration in the US, using the LIWC dictionary. Among those using *non-dictionary methods* are Wang and Tucker [17], who used supervised machine learning models to assign sentiment scores to press releases.

#### Word embedding

One in eight studies applied embedding, mostly word embedding methods. An often-used application of word embedding was mentioned above in "Classification models", where it was used to extend the initial feature set with similar terms in meaning. This method was used in an unsupervised context as well [49] to get a polarization dictionary.

Another inspirational use of the model is exemplified by Brigadir et al. [75], or by Bonikowski et al. [76]: in their case word embedding vector space is used to detect changes in the meaning of certain characteristic terms. Brigadir et al. [75] considered changes in word semantics, both over time and between ideological positions. Bonikowski et al. [76] examined campaign speeches of US presidential candidates Classification models in 2016, by focusing on, for example, the word embedding neighborhood of the term “dangerous,” which illustrates what the candidate views as the most pressing concerns (for example, whereas in case of Trump “refugees” is close to “dangerous” in meaning, in Clinton’s case it is close to “prejudice”).

KhudaBukhsh et al. [14] showed a powerful and interpretable application of word embedding, using word embedding-based machine translation on discussion section of YouTube channels of four prominent US news networks. They showed, that the two sub-communities of CNN and Fox News speak two different languages: what the former label for example, as “biden” and “kkk,” the latter label as “creep” and “blm,” respectively.

Finally, word embedding was also used for user clustering, e.g., Rashed et al. [77] represented users in an embedding space based on their texts, and the representations were then projected onto a lower dimensional space to which cluster analysis was applied.

#### Role of domain knowledge in the analysis

Recent studies, both academic and business, highlighted the importance of building domain knowledge into data science [78, 79]. Domain knowledge is important at each step of a data science project, including research question formulation, data collection, preprocessing, modeling, result interpretation and validation. Therefore, we reviewed whether the authors used domain knowledge at any stage of their research, for example whether they tried to interpret the classification results beyond the assessment of model performance.

If the analysis is specified along substantive questions domain, knowledge is needed to formulate these questions. Stecula and Merkley [80] for example displayed relevant subtleties in the analysis of framing climate change when they defined three types of frames based on previous research. Decadri and Boussalis [81] studied the link between populism/party membership and speech complexity in Italy, with substantive hypotheses, a dictionary of Italian populist rhetoric, and well-chosen metadata.

It is worth noting that in the case of the latter two, the studies were co-authored not only by computer scientist but by domain experts as well. Some examples show that if there are only computer scientists among the authors, the interpretation is often missing, and the research questions are rather technical [82]. And vice versa: if there are only social scientist co-authors, the methodology is fairly simple, although the paper is rich in content: the research questions are explained, the meta-variables are well-chosen, and the results are embedded in an existing scientific discourse [51, 52].

The role of domain knowledge is the most important one at the interpretation step, and especially in supervised classification. Without interpretation, predictive models are black boxes [83]. Understanding *why the model made a certain decision*, and finding the terms that are the most indicative of e.g., conservative versus liberal positions, bring us closer to the understanding of polarization, and help us position the results in the scientific discourse. Three out of five studies that used classification focused only on optimizing classification performance, and did not discuss which linguistic features played a role in the classification [65, 67, 84]. However, there were also articles that went through the interpretation in detail. Diermeier et al. [28] investigated the most distinctive linguistic features in the US Congress, with conclusions such as the one that cultural references are more important than economic references in distinguishing conservative from liberal speeches. Another example is Gentzkow et al. [42], who also very thoroughly went through the interpretation of partisan phrases.

In case of Hirst et al. [33, 34], the main lesson of the paper came from model interpretation: they observed that some of the most distinctive terms of the first analysis (with the Liberal Party as governor) “swapped sides” when turning to the second analysis (with the Conservative Party as governor), which provided evidence that the classifier really picked up features that are related to the government/opposition dimension, instead of political ideology. In other words, the model's real functioning was revealed by the interpretation.

On the interpretability of classification models, it is worth mentioning Praet et al. [86] and Goet [36]. Praet et al. systematically explored the efficiency and interpretability of classification models. Their results showed a clear trade-off between interpretability and discriminative power, e.g., an expert-driven model showed the worst prediction and the best interpretability. According to Goet [36], classifiers ignore dimensionality (contrary to scaling methods), and when we use these models, we sacrifice our ability to make substantive claims about the drivers of polarization. We have seen, however, that the interpretation of the most distinctive words provides some answer to this question, if not as explicitly as in the case of scaling.

It is not only in the case of classification that the question of which words had an impact arises. Medzihorsky et al. [35] used Wordfish to follow the ideological shift of the Republican Party, and identified the most widely used terms and the extent to which they discriminate on the shift dimension. Rashed et al. [77], using cluster analysis, interpreted semantic differences between clusters based on their most prominent terms. Rumshisky et al. [85] gave a detailed interpretation of important “drifter” words that (according to word embedding) changed their meaning the most during the period under examination.

Domain knowledge is also important in the *validation phase* of studies, when the researcher judges the conceptual validity of the results. Goet [36] is an important reference as it provides criteria for evaluating text-based measures of polarization that can be easily followed in practice. According to his criteria, a valid text-based measure, for example, should correspond well to our a-priori expectations, e.g., outliers in our estimates should reflect what we know historically about polarization in the given context. However, validation is completely missing from many of the reviewed papers. Some studies using topic modeling included a validation phase, where the researchers qualitatively assessed the effectiveness of the models [54, 87]. Another way of validation was used in classification studies for example by Taddy [43], Diermeier et al. [27], Bayram et al. [41], Rashed et al. [77], and Gerrish and Blei [39]. Their approach was *to detect the outlier/mispredicted cases*, and to examine them qualitatively. For example, Gerrish and Blei [39], when studying the text of US bills to predict the lawmakers’ vote, detected Rep. Ron Paul as an outlier, and found that he voted more conservatively than expected on healthcare issues. Validation also involves the most critical question that arises when approaching polarization with supervised classification, namely whether ideological differences really underlie the perceived linguistic differences. We will return to this when we discuss causality.

#### The use of qualitative methods

In examining use of qualitative methods, it should be noted that studies using only qualitative text analysis or simple quantitative approach were excluded from the review, so our question was precisely whether a qualitative approach was used in addition to NLP. We have included this issue in our analysis because social research using mixed methods is generally considered to be more valid: it allows for a deeper examination of the phenomenon, supports generalization, increases confidence that findings are not driven by a particular method, and aids interpretation.

Most of the studies reviewed did not use qualitative methods. Some of those that did, used it in the *validation* phase. For example, the validation of topic models requires considerable qualitative work, as the researchers can only assess the interpretability and the effectiveness of the models by actually reading the most relevant texts representing each topic [54, 87]. Yarchi et al. [3] presented a new method that combines NLP with manual content analysis to understand the topics. Taddy [88], on the other hand, is a counterexample, where the model is completely detached from the text, with the analysis not going back to the text during the interpretation of the topics.

Other studies applied a qualitative approach to support *interpretation:* Rho et al. [89] for example used discourse analysis to analyze all comments that contain the top relevant terms computationally detected beforehand. Similarly, Grover et al. [30] carried out a qualitative analysis of tweets containing terms that were found to be important by LIWC analysis.

“*Close reading*,” i.e., a thoughtful interpretation of texts, was explicitly mentioned as the method used by Bonikowski et al. [76] and Sinno et al. [69], for example. In the latter work, close reading was used to understand the motivations behind annotators' decisions.

Dornschneider and Todd [90] was among the few studies in which a *qualitative approach dominated*. They conducted interviews and used sentiment analysis combined with qualitative discourse analysis. In the work of Budak et al. [91], machine learning was used only for technical reasons, to identify the relevant documents, and the essence of the research was given by a close reading of the articles.

#### Addressing causality during the NLP analysis

Though the problem of causation has been extensively studied in empirical social science, it is often neglected in text classification, even though erroneous causal inference does not only concern interpretation, but also compromises the robustness of models.

The most basic causal approach is to refer to timely prevention. E.g., Jensen et al. [12] found that polarized phrases increase in frequency in Google Books before their use increases in congressional speeches. Although they emphasized that causal inference is beyond the scope of their paper, they suggest that their finding is consistent with an autonomous effect of elite discourse on congressional speech.

The most common (even if not explicitly stated) causal problem that arises in the study of ideological polarization is the question of potential confounders, i.e., whether ideological differences are indeed the cause of the detected differences in language use. Very few studies have explicitly mentioned or tried to address this problem. Lin et al. [92] investigated the bitterlemons.org website, which published articles on issues related to the Israeli–Palestinian conflict on a weekly basis with an Israeli and Palestinian editor and guest. The authors applied supervised classification to predict perspective (Israeli or Palestinian). As perspective was better predicted on editors, the authors suggested that there might exist differences between the writing styles of Israeli and Palestinian editors, and it is this that the models had found, not political perspectives. To test this hypothesis, they conducted experiments in which they trained their algorithm on editors and tested it on guests, and vice versa.

Hirst et al. [33, 34] discussed a similar analytic issue. They classified party affiliation across two Canadian Parliaments and revealed that what their models were sensitive to was not expressions of ideology but rather expressions of attack and defense used in a position of opposition and in a position of governing. That is, according to their result, party status (opposition/government) is a confounder when classifying parties.

**Fig. 5:**
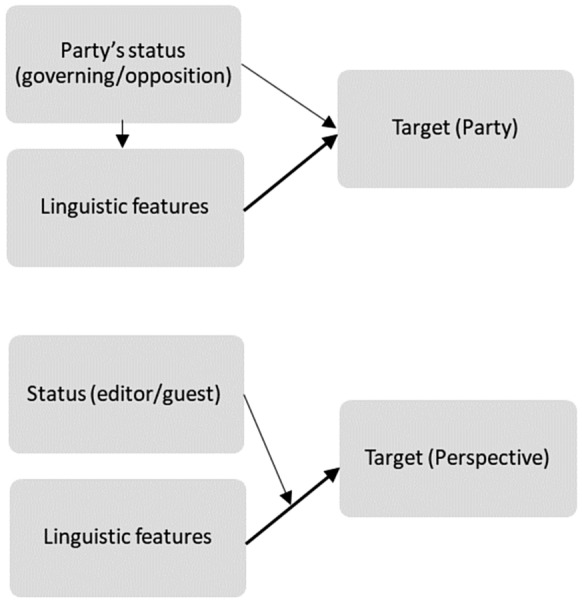
Classification with the presence of a confounder/a moderator. Moderators lie on the causal pathway (bold line), while confounders do not

Both Lin et al. [92] and Hirst et al. [33, 34] suspected a third variable that influenced both the dependent variable and the independent variable, causing a spurious association, but their suspicion was only confirmed in the latter case. In the former case, an effect modification was revealed: only the models’ performance level differed depending on the editor/guest role (the moderator variable), but the same linguistic features distinguished perspectives within both roles. The logic of confounding and effect modification is illustrated in Fig. 5 below.

Very few articles have applied recent approaches to causality. The exceptions include Landeiro and Culotta [93], who use the statistical framework of Judea Pearl, and Widmer et al. [94], who apply the instrumental-variables framework.

## Conclusions

Our review has its limitations. “Political polarization” includes a wide range of definitions, and how “natural language processing” is referred to also varies by disciplines. Although we have attempted to capture these concepts in several different ways, we may have still missed some relevant papers.

Of the initial 3084 hits, we identified 154 relevant studies. The number of papers has risen during the past decade. Most studies focused on the US (*n* = 91), and the cross-national validity of their results has rarely been tested. About 40% (*n* = 66) utilized Twitter data, and one in three studies employed supervised machine learning for predicting ideology/stance.

Some studies demonstrated that the characteristics of political texts depend not only on the political position of their authors, but also on other often-overlooked factors that are not independent of the former (such as the authors’ political engagement, whether their party is in a governing position or in opposition, the texts’ style, genre, or date). Ignoring these factors during data collection, or distinguishing texts based solely on political position, can lead to serious errors, such as overly optimistic association measures, and confounded associations. Some studies suggested that the same issue lies also behind transferability problems, i.e., the corpora that are assumed to be homogeneous are in fact different.

Although the number of studies has grown rapidly in the last years, only a minority of them used domain knowledge to gain insights. Those that did, showed the need for expert interpretation at different points in the analysis (interpreting the most important features, detecting outliers, comparing different models, etc.).

Most studies did not employ the method of close reading, and did not discuss potential problems arising if causal inference is made on textual data. High proportions of studies were non-interdisciplinary in the sense that they were authored without subject matter involvement (45%), or, conversely, they were authored only by social scientists (20%). These observations may be a consequence of the institutionalization of computational research methods outside the social sciences.

However, we have found several inspiring examples for methodological approaches without these shortcomings. We have seen combined use of several types of databases: ones with extra-textual information like user structure, those with metadata of texts, and those that combine polling data with social media data. We have seen that research cover different layers of the political public sphere (politicians, experts, media, or lay public). However, very few studies involved more than one layer, although it can be used to study diffusion processes between the layers.

Many of the points raised by the review are likely to apply to the use of NLP in the social sciences in general, and not just to the study of political polarization. These may include the infrequent use of domain knowledge and mixed methods, the frequent lack of interpretation, the low number of interdisciplinary papers, or the two often conflicting aspects of the data, ease of access and fruitfulness.

Arguments have arisen in recent years that fields adapting artificial intelligence are facing a reproducibility crisis [95]. Among the papers pointing to the causes of the crisis, there were quite a few [5, 96] that argued that the meeting of computer science and applied sciences is more than just adapting large data repositories and tools to analyze them. This meeting also represents a convergence of different fields with different methodological paradigms, and the quality of the research depends on how these paradigms are productively integrated.

We can conclude that the potential of NLP in political polarization research is very high indeed. However, our paper also provides arguments for the integration of explanatory and predictive modeling paradigms, and for a more interdisciplinary approach to polarization research.

## Supplementary Information

Below is the link to the electronic supplementary material.

Supplementary file1 (DOCX 89 KB)

## Data Availability

List of studies included in the review can be found in the supplementary information file.
