# Supplementary material for: A scoping review on the use of natural language processing in research on political polarization: trends and research prospects
Source: J Comput Soc Sci. 2022 Dec 19;6(1):289–313. doi: 10.1007/s42001-022-00196-2 (PMC9762668; doi:10.1007/s42001-022-00196-2)
Supplement: Supplementary file 1 — Supplementary file1 (DOCX 89 KB) [file 42001_2022_196_MOESM1_ESM.docx]

# Supplementary material

## The review’s detailed methodology

We decided to write a scoping review. In addition to aiming for a wider approach, a scoping review, contrary to a systematic review, does not aim to address specific research questions, or to assess the quality of the studies (Arksey and O’Malley 2005). This also determines the logic of the review process: e.g. instead of a definite set of search terms, which cannot be defined, multiple iterations of searches should be used to gradually extend search terms.

An institutional specificity of computer science is that a significant amount of research is never published in journals, therefore we performed the searches using Google Scholar because it indexes journals, books and also conferences and preprint archives. We supplemented the search result list with papers cited in the literature as key sources.

Since the studies we looked for are both defined by their subject matter as well as by their methodology. , our initial search terms were "political polariz(s)ation" AND "natural language processing.” We tried to identify synonyms by checking whether we can find with them the papers we considered relevant. Therefore, we also included e.g. “partisanship” as an alternative to "political polarization.” In terms of methodology: NLP is not a canonicalized term, and the approach is referred to by a different name depending on the research field: text mining in sociology, computation linguistics in linguistics, etc. Our final search terms were as follows:

("political polariz(s)ation" OR "partisan divide" OR "language polariz(s)ation" OR "partisan rhetoric" OR "partisan polariz(s)ation" OR "partisanship" OR "partisan language" OR “polariz(s)ed language” OR “polariz(s)ed rhetoric”)

AND

("natural language processing" OR "text mining" OR "computational linguistics" OR “computational text analysis”).

We got many irrelevant hits, so a range of hard and soft exclusion criteria was defined. According to the hard criteria, non-English language materials, patents, presentation slides, graduate level theses, non-scientific writings, and links to non-functioning pages were excluded.

The soft exclusion criteria aimed at excluding articles with irrelevant content. We excluded papers that just mentioned language polarization without actually studying it. At the same time, we excluded the following areas (which are not directly relevant): studies on language planning, language ideology, echo chambers, and controversy detection. Regarding methodology, we excluded papers using only qualitative text analysis or simple quantitative approaches (e.g. hashtag- or word-frequencies). We included papers that focused primary on algorithmically classifying ideological position from language usage. The two tasks (studying polarization and classifying ideology) are difficult to separate, if only because, as the review showed, the performance of the classification model may serve as a metric of polarization.

Two senior researchers and a research assistant analyzed the relevant studies.

## 1.2 Studies included

| *Authors* | *Title* | *Year* | *Source* | *Publisher* | *URL* | *Cites* |
| --- | --- | --- | --- | --- | --- | --- |
| E Colleoni, A Rozza, A Arvidsson | Echo chamber or public sphere? Predicting political orientation and measuring political homophily in Twitter using big data | 2014 | Journal of Communication | academic.oup.com | https://academic.oup.com/joc/article-abstract/64/2/317/4085994 | 1001 |
| MD Conover, B Gonçalves, J Ratkiewicz, A Flammini, F Menczer | Predicting the political alignment of twitter users | 2011 | 2011 IEEE third … | ieeexplore.ieee.org | https://ieeexplore.ieee.org/abstract/document/6113114/ | 629 |
| J Farrell | Corporate funding and ideological polarization about climate change | 2016 | Proceedings of the National Academy of … | National Acad Sciences | https://www.pnas.org/content/113/1/92.short | 295 |
| R Cohen, D Ruths | Classifying political orientation on Twitter: It's not easy! | 2013 | Proceedings of the International AAAI Conference … | ojs.aaai.org | https://ojs.aaai.org/index.php/ICWSM/article/view/14434 | 254 |
| M Iyyer, P Enns, J Boyd-Graber, P Resnik | Political ideology detection using recursive neural networks | 2014 | … for Computational Linguistics … | aclweb.org | https://www.aclweb.org/anthology/P14-1105.pdf | 244 |
| C Budak, S Goel, JM Rao | Fair and balanced? Quantifying media bias through crowdsourced content analysis | 2016 | Public Opinion Quarterly | academic.oup.com | https://academic.oup.com/poq/article-abstract/80/S1/250/2223443 | 217 |
| M Taddy | Multinomial inverse regression for text analysis | 2013 | Journal of the American Statistical Association | Taylor & Francis | https://www.tandfonline.com/doi/abs/10.1080/01621459.2012.734168 | 207 |
| M Taddy | On estimation and selection for topic models | 2012 | Artificial Intelligence and Statistics | proceedings.mlr.press | http://proceedings.mlr.press/v22/taddy12.html | 180 |
| D Diermeier, JF Godbout, B Yu, S Kaufmann | Language and ideology in Congress | 2012 | British Journal of Political … | cambridge.org | https://www.cambridge.org/core/journals/british-journal-of-political-science/article/language-and-ideology-in-congress/1063F5509BC2ABC3F9A0E164E58157EE | 173 |
| J Chen, G Hsieh, JU Mahmud, J Nichols | Understanding individuals' personal values from social media word use | 2014 | Proceedings of the 17th ACM … | dl.acm.org | https://dl.acm.org/doi/abs/10.1145/2531602.2531608 | 144 |
| BE Lauderdale, A Herzog | Measuring political positions from legislative speech | 2016 | Political Analysis | cambridge.org | https://www.cambridge.org/core/journals/political-analysis/article/measuring-political-positions-from-legislative-speech/35D8B53C4B7367185325C25BBE5F42B4 | 127 |
| S Gerrish, D Blei | How they vote: Issue-adjusted models of legislative behavior | 2012 | Advances in neural information processing … | papers.nips.cc | http://papers.nips.cc/paper/4715-how-they-vote-iss-models-of-legislative-behavior.pdf | 119 |
| P Grover, AK Kar, YK Dwivedi, M Janssen | Polarization and acculturation in US Election 2016 outcomes–Can twitter analytics predict changes in voting preferences | 2019 | Technological Forecasting and … | Elsevier | https://www.sciencedirect.com/science/article/pii/S004016251731421X | 115 |
| J Jensen, S Naidu, E Kaplan, L Wilse-Samson… | Political polarization and the dynamics of political language: Evidence from 130 years of partisan speech [with comments and discussion] | 2012 | Brookings Papers on … | JSTOR | https://www.jstor.org/stable/41825364 | 94 |
| O Tsur, D Calacci, D Lazer | A frame of mind: Using statistical models for detection of framing and agenda setting campaigns | 2015 | … on Natural Language Processing (Volume … | aclweb.org | https://www.cs.cornell.edu/courses/cs6740/2016sp/classes/P15-1157.pdf | 92 |
| J Green, J Edgerton, D Naftel, K Shoub… | Elusive consensus: Polarization in elite communication on the COVID-19 pandemic | 2020 | Science … | advances.sciencemag.org | https://advances.sciencemag.org/content/6/28/eabc2717.abstract | 90 |
| I El Alaoui, Y Gahi, R Messoussi… | A novel adaptable approach for sentiment analysis on big social data | 2018 | … of Big Data | journalofbigdata.springeropen.com | https://journalofbigdata.springeropen.com/articles/10.1186/s40537-018-0120-0 | 72 |
| VA Nguyen, J Boyd-Graber, P Resnik… | Tea party in the house: A hierarchical ideal point topic model and its application to republican legislators in the 112th congress | 2015 | … Computational Linguistics … | aclweb.org | https://www.aclweb.org/anthology/P15-1139.pdf | 63 |
| D Demszky, N Garg, R Voigt, J Zou… | Analyzing polarization in social media: Method and application to tweets on 21 mass shootings | 2019 | arXiv preprint arXiv … | arxiv.org | https://arxiv.org/abs/1904.01596 | 59 |
| V Niculae, C Suen, J Zhang… | Quotus: The structure of political media coverage as revealed by quoting patterns | 2015 | Proceedings of the 24th … | dl.acm.org | https://dl.acm.org/doi/abs/10.1145/2736277.2741688 | 57 |
| AE Boydstun, JH Gross, P Resnik… | Identifying media frames and frame dynamics within and across policy issues | 2013 | New Directions in … | faculty.washington.edu | http://faculty.washington.edu/jwilker/559/frames-2013.pdf | 50 |
| L Rheault, C Cochrane | Word embeddings for the analysis of ideological placement in parliamentary corpora | 2020 | Political Analysis | cambridge.org | https://www.cambridge.org/core/journals/political-analysis/article/word-embeddings-for-the-analysis-of-ideological-placement-in-parliamentary-corpora/017F0CEA9B3DB6E1B94AC36A509A8A7B | 45 |
| A Badawy, A Addawood, K Lerman… | Characterizing the 2016 Russian IRA influence campaign | 2019 | Social Network Analysis … | Springer | https://link.springer.com/article/10.1007/s13278-019-0578-6 | 44 |
| D Fulgoni, J Carpenter, L Ungar… | An empirical exploration of moral foundations theory in partisan news sources | 2016 | Proceedings of the Tenth … | aclweb.org | https://www.aclweb.org/anthology/L16-1591.pdf | 41 |
| K Darwish, P Stefanov, M Aupetit… | Unsupervised user stance detection on twitter | 2020 | Proceedings of the … | ojs.aaai.org | https://ojs.aaai.org/index.php/ICWSM/article/view/7286 | 38 |
| G Hirst, Y Riabinin, J Graham | Party status as a confound in the automatic classification of political speech by ideology | 2010 | Proceedings of the 10th … | www-vhost.cs.toronto.edu | https://www.ledonline.it/ledonline/JADT-2010/allegati/JADT-2010-0731-0742_137-Hirst.pdf | 33 |
| JM Cotelo, FL Cruz, F Enríquez, JA Troyano | Tweet categorization by combining content and structural knowledge | 2016 | Information Fusion | Elsevier | https://www.sciencedirect.com/science/article/pii/S1566253516000099 | 32 |
| DA Stecula, E Merkley | Framing climate change: economics, ideology, and uncertainty in American news media content from 1988 to 2014 | 2019 | Frontiers in Communication | frontiersin.org | https://www.frontiersin.org/articles/10.3389/fcomm.2019.00006/full | 31 |
| V Kulkarni, J Ye, S Skiena, WY Wang | Multi-view models for political ideology detection of news articles | 2018 | arXiv preprint arXiv:1809.03485 | arxiv.org | https://arxiv.org/abs/1809.03485 | 30 |
| I Brigadir, D Greene, P Cunningham | Analyzing discourse communities with distributional semantic models | 2015 | Proceedings of the ACM Web … | dl.acm.org | https://dl.acm.org/doi/abs/10.1145/2786451.2786470?casa_token=jfJp4BnNS1AAAAAA:SyfZoMNCTHPx7PpMm8gFQGWXJu0mxSRotut4ChT8XFa3BrkV18ioOrBvoWL4qleZ2y8u6ryIY92atA | 27 |
| Y Wang, Y Feng, Z Hong, R Berger, J Luo | How polarized have we become? a multimodal classification of trump followers and clinton followers | 2017 | International Conference on … | Springer | https://link.springer.com/chapter/10.1007/978-3-319-67217-5_27 | 27 |
| E Tzelgov | Cross-cutting issues, intraparty dissent and party strategy: The issue of European integration in the House of Commons | 2014 | European Union Politics | journals.sagepub.com | https://journals.sagepub.com/doi/abs/10.1177/1465116513509307 | 25 |
| J An, H Kwak, O Posegga, A Jungherr | Political discussions in homogeneous and cross-cutting communication spaces | 2019 | Proceedings of the International … | ojs.aaai.org | https://ojs.aaai.org/index.php/ICWSM/article/view/3210 | 24 |
| R Baly, G Karadzhov, A Saleh, J Glass… | Multi-task ordinal regression for jointly predicting the trustworthiness and the leading political ideology of news media | 2019 | arXiv preprint arXiv … | arxiv.org | https://arxiv.org/abs/1904.00542 | 24 |
| B Kelly, A Manela, A Moreira | Text selection | 2021 | Journal of Business & Economic … | Taylor & Francis | https://www.tandfonline.com/doi/abs/10.1080/07350015.2021.1947843 | 22 |
| M Lai, C Bosco, V Patti, D Virone | Debate on political reforms in Twitter: A hashtag-driven analysis of political polarization | 2015 | 2015 IEEE International … | ieeexplore.ieee.org | https://ieeexplore.ieee.org/abstract/document/7344884/ | 22 |
| D Hu, S Jiang, R E. Robertson, C Wilson | Auditing the partisanship of google search snippets | 2019 | The World Wide Web … | dl.acm.org | https://dl.acm.org/doi/abs/10.1145/3308558.3313654 | 21 |
| MZ Ansari, MB Aziz, MO Siddiqui, H Mehra… | Analysis of political sentiment orientations on twitter | 2020 | Procedia Computer … | Elsevier | https://www.sciencedirect.com/science/article/pii/S1877050920306669 | 20 |
| S Xu, A Zhou | Hashtag homophily in twitter network: Examining a controversial cause-related marketing campaign | 2020 | Computers in human behavior | Elsevier | https://www.sciencedirect.com/science/article/pii/S0747563219302936 | 20 |
| G Hirst, Y Riabinin, J Graham… | Text to Ideology or Text to Party Status? | 2014 | From text to political … | books.google.com | https://books.google.com/books?hl=en&lr=&id=R6diAwAAQBAJ&oi=fnd&pg=PA93&dq=%22partisanship%22%7C%22partisan+language%22%7C%22political+polarisation%22%7C%22language+polarisation%22%7C%22partisan+polarisation+and%22+%22natural+language+processing%22%7C%22text+mining%22%7C%22computational+linguistics%22%7C%22computational+text+analysis%22&ots=wNVIqer8R-&sig=qIcEE92lFBhT-jN6tGH_3G4FldU | 19 |
| H Takikawa, K Nagayoshi | Political polarization in social media: Analysis of the “Twitter political field” in Japan | 2017 | … conference on big data (big data) | ieeexplore.ieee.org | https://ieeexplore.ieee.org/abstract/document/8258291/ | 19 |
| SS Bodrunova, I Blekanov… | Beyond left and right: real-world political polarization in Twitter discussions on inter-ethnic conflicts | 2019 | Media and … | refubium.fu-berlin.de | https://refubium.fu-berlin.de/handle/fub188/25967 | 19 |
| JP Gauvin, C Chhim, M Medeiros | Did they mind the gap? Voter/party ideological proximity between the BQ, the NDP and Quebec voters, 2006–2011 | 2016 | Canadian Journal of Political … | cambridge.org | https://www.cambridge.org/core/journals/canadian-journal-of-political-science-revue-canadienne-de-science-politique/article/did-they-mind-the-gap-voterparty-ideological-proximity-between-the-bq-the-ndp-and-quebec-voters-20062011/67A3C8C199A8F3F061F4982D9F4891F9 | 18 |
| M Lai, M Tambuscio, V Patti, G Ruffo… | Stance polarity in political debates: A diachronic perspective of network homophily and conversations on Twitter | 2019 | Data & Knowledge … | Elsevier | https://www.sciencedirect.com/science/article/pii/S0169023X19300187 | 18 |
| D Karamshuk, T Lokot, O Pryymak, N Sastry | Identifying partisan slant in news articles and twitter during political crises | 2016 | International Conference on … | Springer | https://link.springer.com/chapter/10.1007/978-3-319-47880-7_16 | 17 |
| EHR Rho, G Mark, M Mazmanian | Fostering civil discourse online: Linguistic behavior in comments of# metoo articles across political perspectives | 2018 | Proceedings of the ACM on Human … | dl.acm.org | https://dl.acm.org/doi/abs/10.1145/3274416 | 17 |
| L Fan, M White, E Sharma, R Su, PK Choubey… | In plain sight: Media bias through the lens of factual reporting | 2019 | arXiv preprint arXiv … | arxiv.org | https://arxiv.org/abs/1909.02670 | 17 |
| L Hemphill, A Culotta, M Heston | # Polar Scores: Measuring partisanship using social media content | 2016 | Journal of Information … | Taylor & Francis | https://www.tandfonline.com/doi/abs/10.1080/19331681.2016.1214093 | 17 |
| T Thonet, G Cabanac, M Boughanem… | Users are known by the company they keep: Topic models for viewpoint discovery in social networks | 2017 | Proceedings of the … | dl.acm.org | https://dl.acm.org/doi/abs/10.1145/3132847.3132897?casa_token=FJD9sMrrhwUAAAAA:_TIOgk7saXoJnbaAh4nmqOd1b8GnTlsqMQ1WZe5wjwFpB8aFJzzdqVxR2UcT3TBsYFdN5bGexAmkBw | 17 |
| Z Jelveh, B Kogut, S Naidu | Detecting latent ideology in expert text: Evidence from academic papers in economics | 2014 | … methods in natural language processing … | aclweb.org | https://www.aclweb.org/anthology/D14-1191.pdf | 17 |
| F Giglietto, L Iannelli, L Rossi, A Valeriani… | Mapping italian news media political coverage in the lead-up to 2018 general election | 2018 | Available at SSRN … | papers.ssrn.com | https://papers.ssrn.com/sol3/papers.cfm?abstract_id=3179930 | 16 |
| J Medzihorsky, L Littvay, EK Jenne | Has the tea party era radicalized the republican party? evidence from text analysis of the 2008 and 2012 republican primary debates | 2014 | PS: Political Science & Politics | cambridge.org | https://www.cambridge.org/core/journals/ps-political-science-and-politics/article/has-the-tea-party-era-radicalized-the-republican-party-evidence-from-text-analysis-of-the-2008-and-2012-republican-primary-debates/D0836CD67B027F3D31C7401ACA4F9183 | 16 |
| M Yarchi, C Baden, N Kligler-Vilenchik | Political polarization on the digital sphere: A cross-platform, over-time analysis of interactional, positional, and affective polarization on social media | 2021 | Political Communication | Taylor & Francis | https://www.tandfonline.com/doi/abs/10.1080/10584609.2020.1785067 | 16 |
| Q Shen, C Rose | The discourse of online content moderation: Investigating polarized user responses to changes in reddit's quarantine policy | 2019 | Proceedings of the Third Workshop on Abusive … | aclweb.org | https://www.aclweb.org/anthology/W19-3507.pdf | 16 |
| M Gross, M Jankowski | Dimensions of political conflict and party positions in multi-level democracies: evidence from the Local Manifesto Project | 2020 | West European Politics | Taylor & Francis | https://www.tandfonline.com/doi/abs/10.1080/01402382.2019.1602816 | 15 |
| A Trabelsi, O Zaiane | Unsupervised model for topic viewpoint discovery in online debates leveraging author interactions | 2018 | Proceedings of the International AAAI Conference … | ojs.aaai.org | https://ojs.aaai.org/index.php/ICWSM/article/view/15021 | 14 |
| A Aldayel, W Magdy | Your stance is exposed! analysing possible factors for stance detection on social media | 2019 | Proceedings of the ACM on Human-Computer … | dl.acm.org | https://dl.acm.org/doi/abs/10.1145/3359307 | 13 |
| J Wihbey, K Joseph, D Lazer | The social silos of journalism? Twitter, news media and partisan segregation | 2019 | New Media & Society | journals.sagepub.com | https://journals.sagepub.com/doi/abs/10.1177/1461444818807133 | 13 |
| M Boireau | Determining political stances from twitter timelines: The belgian parliament case | 2014 | Proceedings of the 2014 Conference on Electronic … | dl.acm.org | https://dl.acm.org/doi/abs/10.1145/2729104.2729114 | 13 |
| A Samantray, P Pin | Credibility of climate change denial in social media | 2019 | Palgrave Communications | nature.com | https://www.nature.com/articles/s41599-019-0344-4 | 11 |
| CJ Montiel, J Uyheng | Mapping contentious collective emotions in a populist democracy: Duterte's push for philippine federalism | 2020 | Political Psychology | Wiley Online Library | https://onlinelibrary.wiley.com/doi/abs/10.1111/pops.12648 | 11 |
| K Darwish | Quantifying polarization on twitter: the kavanaugh nomination | 2019 | International Conference on Social Informatics | Springer | https://link.springer.com/chapter/10.1007/978-3-030-34971-4_13 | 11 |
| DE Pozen, EL Talley, J Nyarko | A computational analysis of constitutional polarization | 2019 | Cornell L. Rev. | HeinOnline | https://heinonline.org/hol-cgi-bin/get_pdf.cgi?handle=hein.journals/clqv105&section=4 | 10 |
| H Karimi, T Derr, A Brookhouse, J Tang | Multi-factor congressional vote prediction | 2019 | Proceedings of the 2019 IEEE … | dl.acm.org | https://dl.acm.org/doi/abs/10.1145/3341161.3342884 | 10 |
| S Jordan, CML Webb, BD Wood | The president, polarization and the party platforms, 1944–2012 | 2014 | The Forum | degruyter.com | https://www.degruyter.com/document/doi/10.1515/for-2014-0024/html | 10 |
| G Gorrell, M Greenwood, I Roberts, D Maynard… | Online abuse of uk mps in 2015 and 2017: Perpetrators, targets, and topics | 2018 | arXiv preprint arXiv … | arxiv.org | https://arxiv.org/abs/1804.01498 | 9 |
| JH Gross, B Acree, Y Sim, NA Smith | Testing the etch-a-sketch hypothesis: a computational analysis of Mitt Romney's ideological makeover during the 2012 primary vs. general elections | 2013 | APSA 2013 Annual Meeting … | papers.ssrn.com | https://papers.ssrn.com/sol3/papers.cfm?abstract_id=2299991 | 9 |
| T Grover, E Bayraktaroglu, G Mark… | Moral and affective differences in us immigration policy debate on twitter | 2019 | … Cooperative Work (CSCW) | Springer | https://link.springer.com/article/10.1007/s10606-019-09357-w | 9 |
| A Zubiaga, B Wang, M Liakata, R Procter | Stance classification of social media users in independence movements | 2017 | Catalonia | researchgate.net | https://www.researchgate.net/profile/Rob-Procter/publication/314093496_Stance_Classification_of_Social_Media_Users_in_Independence_Movements/links/595ce309a6fdcc8623299a14/Stance-Classification-of-Social-Media-Users-in-Independence-Movements.pdf | 8 |
| IR Iliev, X Huang, YR Gel | Political rhetoric through the lens of non‐parametric statistics: are our legislators that different? | 2019 | Journal of the Royal Statistical … | Wiley Online Library | https://rss.onlinelibrary.wiley.com/doi/abs/10.1111/rssa.12421 | 8 |
| J Sanders, G Lisi, C Schonhardt-Bailey | Themes and topics in parliamentary oversight hearings: a new direction in textual data analysis | 2017 | Statistics, Politics and Policy | degruyter.com | https://www.degruyter.com/document/doi/10.1515/spp-2017-0012/html | 8 |
| L Belcastro, R Cantini, F Marozzo, D Talia… | Learning political polarization on social media using neural networks | 2020 | IEEE Access | ieeexplore.ieee.org | https://ieeexplore.ieee.org/abstract/document/9026882/ | 8 |
| M Quraishi, P Fafalios, E Herder | Viewpoint discovery and understanding in social networks | 2018 | … of the 10th ACM Conference on Web … | dl.acm.org | https://dl.acm.org/doi/abs/10.1145/3201064.3201076?casa_token=1OB-RUbr2mYAAAAA:php72bzgADCdbpUJh1VaccrM0DQkyC0ZrsS4rTa89MsLrffelTqKQ6VO39sa3RJbLzAlLuhMW8aqtQ | 8 |
| P Stefanov, K Darwish, A Atanasov, P Nakov | Predicting the topical stance of media and popular twitter users | 2019 | arXiv preprint arXiv … | arxiv.org | https://arxiv.org/abs/1907.01260 | 8 |
| A Rumshisky, M Gronas, P Potash, M Dubov… | Combining network and language indicators for tracking conflict intensity | 2017 | … Conference on Social … | Springer | https://link.springer.com/chapter/10.1007/978-3-319-67256-4_31 | 7 |
| AR KhudaBukhsh, R Sarkar, MS Kamlet… | We Don't Speak the Same Language: Interpreting Polarization through Machine Translation | 2020 | arXiv preprint arXiv … | arxiv.org | https://arxiv.org/abs/2010.02339 | 7 |
| CI Hausladen, MH Schubert, E Ash | Text classification of ideological direction in judicial opinions | 2020 | International Review of Law and … | Elsevier | https://www.sciencedirect.com/science/article/pii/S0144818819303667 | 7 |
| S Roy, D Goldwasser | Weakly supervised learning of nuanced frames for analyzing polarization in news media | 2020 | arXiv preprint arXiv:2009.09609 | arxiv.org | https://arxiv.org/abs/2009.09609 | 7 |
| A Bilbao-Jayo, A Almeida | Political discourse classification in social networks using context sensitive convolutional neural networks | 2018 | … Workshop on Natural Language Processing … | aclweb.org | https://www.aclweb.org/anthology/W18-3513.pdf | 6 |
| M Romenskyy, V Spaiser, T Ihle… | Polarized Ukraine 2014: opinion and territorial split demonstrated with the bounded confidence XY model, parametrized by Twitter data | 2018 | Royal Society open … | royalsocietypublishing.org | https://royalsocietypublishing.org/doi/abs/10.1098/rsos.171935 | 6 |
| MR Haupt, A Jinich-Diamant, J Li, M Nali… | Characterizing twitter user topics and communication network dynamics of the “liberate” movement during COVID-19 using unsupervised machine learning and social … | 2021 | Online Social Networks … | Elsevier | https://www.sciencedirect.com/science/article/pii/S2468696420300550 | 6 |
| T Kobayashi, Y Ogawa, T Suzuki… | News audience fragmentation in the Japanese Twittersphere | 2019 | Asian Journal of … | Taylor & Francis | https://www.tandfonline.com/doi/abs/10.1080/01292986.2018.1458326 | 6 |
| V Landeiro, A Culotta | Robust text classification under confounding shift | 2018 | Journal of Artificial Intelligence Research | jair.org | https://www.jair.org/index.php/jair/article/view/11248 | 6 |
| Y Freire-Vidal, E Graells-Garrido | Characterization of local attitudes toward immigration using social media | 2019 | … Proceedings of The 2019 World Wide … | dl.acm.org | https://dl.acm.org/doi/abs/10.1145/3308560.3316455 | 6 |
| BDL Acree, JH Gross, NA Smith… | Etch-a-Sketching: Evaluating the post-primary rhetorical moderation hypothesis | 2020 | American Politics … | journals.sagepub.com | https://journals.sagepub.com/doi/abs/10.1177/1532673X18800017 | 5 |
| C Koylu, R Larson, BJ Dietrich… | CarSenToGram: geovisual text analytics for exploring spatiotemporal variation in public discourse on Twitter | 2019 | Cartography and … | Taylor & Francis | https://www.tandfonline.com/doi/abs/10.1080/15230406.2018.1510343 | 5 |
| C Shurafa, K Darwish, W Zaghouani | Political Framing: US COVID19 Blame Game | 2020 | International Conference on Social … | Springer | https://link.springer.com/chapter/10.1007/978-3-030-60975-7_25 | 5 |
| D Röchert, G Neubaum, B Ross, F Brachten… | Opinion-based Homogeneity on YouTube: Combining Sentiment and Social Network Analysis | 2020 | Computational … | aup-online.com | https://www.aup-online.com/content/journals/10.5117/CCR2020.1.004.ROCH | 5 |
| H Yan, A Lavoie, S Das | The perils of classifying political orientation from text | 2017 | LINKDEM@ IJCAI | openreview.net | https://openreview.net/pdf?id=H14FuNM_-S | 5 |
| R Baly, GDS Martino, J Glass, P Nakov | We Can Detect Your Bias: Predicting the Political Ideology of News Articles | 2020 | arXiv preprint arXiv:2010.05338 | arxiv.org | https://arxiv.org/abs/2010.05338 | 5 |
| S Decadri, C Boussalis | Populism, party membership, and language complexity in the Italian chamber of deputies | 2020 | Journal of Elections, Public Opinion and … | Taylor & Francis | https://www.tandfonline.com/doi/abs/10.1080/17457289.2019.1593182 | 5 |
| A Rao, F Morstatter, M Hu, E Chen, K Burghardt… | Political Partisanship and Anti-Science Attitudes in Online Discussions about Covid-19 | 2020 | arXiv preprint arXiv … | arxiv.org | https://arxiv.org/abs/2011.08498 | 4 |
| B Bonikowski, Y Feinstein… | The Polarization of Nationalist Cleavages and the 2016 US Presidential Election | 2019 | Annual meeting of … | ucrpoliticaleconomy.ucr.edu | https://ucrpoliticaleconomy.ucr.edu/wp-content/uploads/2019/04/Bonikowski-Feinstein-and-Bock-Polarization-of-Nationalist-Cleavages-UC-Riverside.pdf | 4 |
| E Ademmer, T Stöhr | The making of a new cleavage? Evidence from social media debates about migration | 2019 |  | econstor.eu | https://www.econstor.eu/handle/10419/204498 | 4 |
| E Ash, DL Chen, W Lu | Polarization of US Circuit Court judges: a machine learning approach | 2017 | Available at SSRN 2993009 | papers.ssrn.com | https://papers.ssrn.com/sol3/Delivery.cfm?abstractid=2993009 | 4 |
| E Sapiro-Gheiler | " Read My Lips": Using Automatic Text Analysis to Classify Politicians by Party and Ideology | 2018 | arXiv preprint arXiv:1809.00741 | arxiv.org | https://arxiv.org/abs/1809.00741 | 4 |
| GR Mendez, AG Cosby, SD Mohanty | Obamacare on Twitter: Online Political Participation and its Effects on Polarisation | 2018 | Teorija in Praksa | researchgate.net | https://www.researchgate.net/profile/Gina_Rico_Mendez/publication/326326729_Obamacare_on_Twitter_Online_Political_Participation_and_its_Effects_on_Political_Polarisation/links/5b461324a6fdcc6619183829/Obamacare-on-Twitter-Online-Political-Participation-and-its-Effects-on-Political-Polarisation.pdf | 4 |
| JM Robles, D Velez, S De Marco… | Affective homogeneity in the Spanish general election debate. A comparative analysis of social networks political agents | 2020 | Information … | Taylor & Francis | https://www.tandfonline.com/doi/abs/10.1080/1369118X.2018.1499792 | 4 |
| L Belcastro, R Cantini, F Marozzo… | Discovering political polarization on social media: A case study | 2019 | … Knowledge and Grids … | ieeexplore.ieee.org | https://ieeexplore.ieee.org/abstract/document/9044124/ | 4 |
| P Widmer, S Galletta, E Ash | Media Slant is Contagious | 2020 | Center for Law & … | research-collection.ethz.ch | https://www.research-collection.ethz.ch/bitstream/handle/20.500.11850/454192/1/CLE_WP_2020_14.pdf | 4 |
| S Martin-Gutierrez, JC Losada… | Semi-automatic training set construction for supervised sentiment analysis in political contexts | 2018 | 2018 IEEE/ACM … | ieeexplore.ieee.org | https://ieeexplore.ieee.org/abstract/document/8508386/ | 4 |
| B Yu, D Diermeier | A longitudinal study of language and ideology in congress | 2010 |  | surface.syr.edu | https://surface.syr.edu/istpub/35/ | 3 |
| CG Chiru, T Dimcica… | Expression of Political Opinions in Press | 2017 | 2017 21st International … | ieeexplore.ieee.org | https://ieeexplore.ieee.org/abstract/document/7968560/ | 3 |
| G Gennaro, E Ash | Emotion and Reason in Political Language | 2021 | Center for Law & Economics … | research-collection.ethz.ch | https://www.research-collection.ethz.ch/bitstream/handle/20.500.11850/468192/1/CLE_WP_2021_02.pdf | 3 |
| H Yan, S Das, A Lavoie, S Li, B Sinclair | The Congressional Classification Challenge: Domain Specificity and Partisan Intensity | 2019 | Proceedings of the 2019 ACM … | dl.acm.org | https://dl.acm.org/doi/abs/10.1145/3328526.3329582 | 3 |
| IJ Serrano-Contreras, J García-Marín… | Measuring online political dialogue: does polarization trigger more deliberation? | 2020 | Media and … | ssoar.info | https://www.ssoar.info/ssoar/handle/document/70408 | 3 |
| MN Habibi | Analysis of Indonesia Politics Polarization before 2019 President Election Using Sentiment Analysis and Social Network Analysis. | 2019 | International Journal of Modern Education & … | mecs-press.org | http://www.mecs-press.org/ijmecs/ijmecs-v11-n11/IJMECS-V11-N11-4.pdf | 3 |
| S Dornschneider, J Todd | Everyday sentiment among unionists and nationalists in a Northern Irish town | 2020 | Irish Political Studies | Taylor & Francis | https://www.tandfonline.com/doi/abs/10.1080/07907184.2020.1743023 | 3 |
| A Rashed, M Kutlu, K Darwish, T Elsayed… | Embeddings-Based Clustering for Target Specific Stances: The Case of a Polarized Turkey | 2020 | arXiv preprint arXiv … | arxiv.org | https://arxiv.org/abs/2005.09649 | 2 |
| M Kutlu, K Darwish, C Bayrak, A Rashed… | Embedding-based qualitative analysis of polarization in turkey | 2019 | arXiv preprint arXiv … | arxiv.org | https://arxiv.org/abs/1909.10213 | 2 |
| MG Søyland, E Lapponi | Party polarization and parliamentary speech | 2017 | Working paper | researchgate.net | https://www.researchgate.net/profile/Martin_Soyland/publication/319529973_Party_Polarization_and_Parliamentary_Speech/links/59b17168aca2728472d12f82/Party-Polarization-and-Parliamentary-Speech.pdf | 2 |
| MZ Ansari, AF Siddiqui, M Anas | Inferring Political Preferences from Twitter | 2020 | arXiv preprint arXiv:2007.10604 | arxiv.org | https://arxiv.org/abs/2007.10604 | 2 |
| PY Wu, WR Mebane Jr, L Woods… | Partisan associations of Twitter users based on their self-descriptions and word embeddings | 2019 | … Annual Meeting of … | www-personal.umich.edu | http://www-personal.umich.edu/~wmebane/partisanassociations_wumebanewoodsklaverdue_apsa2019.pdf | 2 |
| R Liu, C Jia, S Vosoughi | A transformer-based framework for neutralizing and reversing the political polarity of news articles | 2021 | Proceedings of the ACM on Human-Computer … | dl.acm.org | https://dl.acm.org/doi/abs/10.1145/3449139 | 2 |
| R Liu, L Wang, C Jia, S Vosoughi | Political Depolarization of News Articles Using Attribute-aware Word Embeddings | 2021 | arXiv preprint arXiv:2101.01391 | arxiv.org | https://arxiv.org/abs/2101.01391 | 2 |
| S Appelrouth | Envisioning America and the American Self: Republican and Democratic Party Platforms, 1840–2016 | 2019 |  | taylorfrancis.com | https://www.taylorfrancis.com/books/mono/10.4324/9781315107738/envisioning-america-american-self-scott-appelrouth | 2 |
| T Coutto | Half-full or half-empty? Framing of UK–EU relations during the Brexit referendum campaign | 2020 | Journal of European Integration | Taylor & Francis | https://www.tandfonline.com/doi/abs/10.1080/07036337.2020.1792465 | 2 |
| U Bayram, J Pestian, D Santel… | What's in a Word? Detecting Partisan Affiliation from Word Use in Congressional Speeches | 2019 | 2019 International Joint … | ieeexplore.ieee.org | https://ieeexplore.ieee.org/abstract/document/8851739/ | 2 |
| A Simchon, WJ Brady, JJ Van Bavel | Troll and Divide: The Language of Online Polarization | 2020 |  | psyarxiv.com | https://psyarxiv.com/xjd64/download?format=pdf | 1 |
| B Acree | Deep Learning and Ideological Rhetoric | 2016 |  | cdr.lib.unc.edu | https://cdr.lib.unc.edu/concern/dissertations/s1784m14f | 1 |
| D Warmsley, J Xu, TC Lu | From Gamergate to FIFA: Identifying Polarized Groups in Online Social Media | 2018 | … Conference on Big Data (Big Data) | ieeexplore.ieee.org | https://ieeexplore.ieee.org/abstract/document/8621869/ | 1 |
| E Jing, YY Ahn | Characterizing Partisan Political Narratives about COVID-19 on Twitter | 2021 | arXiv preprint arXiv:2103.06960 | arxiv.org | https://arxiv.org/abs/2103.06960 | 1 |
| M Lai, V Patti, G Ruffo, P Rosso | # Brexit: Leave or remain? the role of user's community and diachronic evolution on stance detection | 2020 | Journal of Intelligent & Fuzzy … | content.iospress.com | https://content.iospress.com/articles/journal-of-intelligent-and-fuzzy-systems/ifs179895 | 1 |
| ND Goet | Measuring Polarisation with Text Analysis | 2017 |  | democratic-anxieties.eu | https://www.democratic-anxieties.eu/wordpress/wp-content/uploads/2017/10/Goet_Berlin2017.pdf | 1 |
| S Kumar, RV Cox, M Babcock, KM Carley | A Weakly Supervised Approach for Classifying Stance in Twitter Replies | 2021 | arXiv preprint arXiv … | arxiv.org | https://arxiv.org/abs/2103.07098 | 1 |
| SC Guntuku, J Purtle, ZF Meisel, RM Merchant… | Partisan Differences in Twitter Language Among US Legislators During the COVID-19 Pandemic: Cross-sectional Study | 2021 | Journal of Medical … | jmir.org | https://www.jmir.org/2021/6/e27300 | 1 |
| V Morini, L Pollacci, G Rossetti | Capturing Political Polarization of Reddit Submissions in the Trump Era. | 2020 | SEBD | aiucd2021.labcd.unipi.it | https://aiucd2021.labcd.unipi.it/wp-content/uploads/2021/01/a068.pdf | 1 |
| A Budhiraja, J Pal | Twitter and political culture: Short text embeddings as a window into political fragmentation | 2020 | Proceedings of the 3rd ACM SIGCAS Conference on … | dl.acm.org | https://dl.acm.org/doi/abs/10.1145/3378393.3402276?casa_token=4C-rD5gNEbQAAAAA:2oaNztKqvtssSgJWTbFQbq4fTK2eyoXHfLfsK40F1zi3coSq0u-sYpLZcQYHuSCRNIpMs1y0NBGZxQ | 0 |
| B Bonikowski, Y Feinstein, S Bock | The Partisan Sorting ofamerica': How Nationalist Cleavages Shaped the 2016 US Presidential Election | 2019 |  | osf.io | https://osf.io/preprints/socarxiv/pmg95/ | 0 |
| B Sinno, B Oviedo, K Atwell, M Alikhani… | Political Ideology and Polarization of Policy Positions: A Multi-dimensional Approach | 2021 | arXiv preprint arXiv … | arxiv.org | https://arxiv.org/abs/2106.14387 | 0 |
| C Le Pennec | Strategic Campaign Communication: Evidence from 30,000 Candidate Manifestos | 2020 |  | soda-wps.s3.amazonaws.com | http://soda-wps.s3.amazonaws.com/RePEc/ajr/sodwps/2020-05.pdf | 0 |
| D Domalewska | Disinformation and Polarization in the Online Debate During the 2020 Presidential Election in Poland | 2021 | Safety & Defense | sd-magazine.eu | https://www.sd-magazine.eu/index.php/sd/article/view/92 | 0 |
| EC Tucker, CJ Capps, L Shamir | A data science approach to 138 years of congressional speeches | 2020 | Heliyon | Elsevier | https://www.sciencedirect.com/science/article/pii/S2405844020312615 | 0 |
| G Rossetti | Capturing Political Polarization of Reddit Submissions in the Trump Era | 2020 |  | ceur-ws.org | http://ceur-ws.org/Vol-2646/39-paper.pdf | 0 |
| G Spell, B Guay, S Hillygus, L Carin | An Embedding Model for Estimating Legislative Preferences from the Frequency and Sentiment of Tweets | 2020 | … in Natural Language Processing … | aclweb.org | https://www.aclweb.org/anthology/2020.emnlp-main.46.pdf | 0 |
| J Gelman, SL Wilson | Measuring Congressional Partisanship and Its Consequences | 2021 | Legislative Studies Quarterly | Wiley Online Library | https://onlinelibrary.wiley.com/doi/abs/10.1111/lsq.12331 | 0 |
| LA Cornelissen, C de Bruyn, MK Ledingwane… | Cross-Sample Community Detection and Sentiment Analysis: South African Twitter | 2019 | Proceedings of the … | dl.acm.org | https://dl.acm.org/doi/abs/10.1145/3351108.3351135 | 0 |
| M Cardaioli, P Kaliyar, P Capuozzo… | Predicting Twitter Users' Political Orientation: An Application to the Italian Political Scenario | 2020 | 2020 IEEE/ACM … | ieeexplore.ieee.org | https://ieeexplore.ieee.org/abstract/document/9381470/ | 0 |
| M Søyland | Multi-party Classification of Parliamentary Debates: intra-party cohesion and Inter-Party Relations Measured in Text | 2020 |  | researchgate.net | https://www.researchgate.net/profile/Martin-Soyland/publication/344557289_Multi-party_Classification_of_Parliamentary_Debates_Intra-party_cohesion_and_Inter-Party_Relations_Measured_in_Text/links/5f80384692851c14bcb92fd7/Multi-party-Classification-of-Parliamentary-Debates-Intra-party-cohesion-and-Inter-Party-Relations-Measured-in-Text.pdf | 0 |
| R Villa-Cox, AR KhudaBukhsh, KM Carley | Exploring Polarization of Users Behavior on Twitter During the 2019 South American Protests | 2021 | arXiv preprint arXiv … | arxiv.org | https://arxiv.org/abs/2104.05611 | 0 |
| RT Wang, PD Tucker | How Partisanship Influences What Congress Says Online and How They Say It | 2021 | American Politics Research | journals.sagepub.com | https://journals.sagepub.com/doi/abs/10.1177/1532673X20939498 | 0 |
| S Diaf, J Döpke, U Fritsche, I Rockenbach | Sharks and minnows in a shoal of words: Measuring latent ideological positions of German economic research institutes based on text mining techniques | 2020 |  | econstor.eu | https://www.econstor.eu/handle/10419/225085 | 0 |
| S Garcia, HF Mueller, C Sanz | Economic Uncertainty and Divisive Politics: Evidence from the | 2020 |  | papers.ssrn.com | https://bse.eu/research/working-papers/economic-uncertainty-and-divisive-politics-evidence-dos-espanas | 0 |
| S Praet, P Van Aelst, W Daelemans… | Comparing automated content analysis methods to distinguish issue communication by political parties on Twitter | 2021 | Available at SSRN … | papers.ssrn.com | https://papers.ssrn.com/sol3/papers.cfm?abstract_id=3782027 | 0 |
| T Strzalkowski, A Newheiser, N Kemper, N Sa… | Generating Ethnographic Models from Communities' Online Data | 2020 | Proceedings of the … | aclweb.org | https://www.aclweb.org/anthology/2020.figlang-1.23/ | 0 |
| V Barash, P Kolozaridi, D Muravyov… | Evolution of the Digitally Mediated Public Sphere in Russia, 2012-2018 | 2020 | Available at SSRN … | papers.ssrn.com | https://papers.ssrn.com/sol3/papers.cfm?abstract_id=3740730 | 0 |
| Y Samih, K Darwish | A Few Topical Tweets are Enough for Effective User-Level Stance Detection | 2020 | arXiv preprint arXiv:2004.03485 | arxiv.org | https://arxiv.org/abs/2004.03485 | 0 |
| Z He, N Mokhberian, A Camara, A Abeliuk… | Detecting Polarized Topics in COVID-19 News Using Partisanship-aware Contextualized Topic Embeddings | 2021 | arXiv preprint arXiv … | arxiv.org | https://arxiv.org/abs/2104.07814 | 0 |
| Gentzkow, M., Shapiro, J.M., Taddy, M. | Measuring Group Differences in High-Dimensional Choices: Method and Application to Congressional Speech | 2019 | Econometrica | | https://onlinelibrary.wiley.com/doi/full/10.3982/ECTA16566 | These papers were manually added |
| Hofmann, K., Marakasova, A., Baumann, A., Neidhardt, J., Wissik, T. | Comparing Lexical Usage in Political Discourse across Diachronic Corpora | 2020 | Proceedings of ParlaCLARIN II Workshop | | https://www.aclweb.org/anthology/2020.parlaclarin-1.11.pdf |  |
| Lin, W.-H., Wilson, Th, Wiebe, J., Hauptmann, A. | Which Side are You on? Identifying Perspectives at the Document and Sentence Levels | 2006 | Proceedings of the 10th Conference on Computational Natural Language Learning | | https://aclanthology.org/W06-2915.pdf |  |
| Dehghani, M., Gratch, J., Sachdeva, S., Sagae, K. | Analyzing Conservative and Liberal Blogs Related to the Construction of the ‘Ground Zero Mosque’ | 2011 | Proceedings of the Annual Meeting of the Cognitive Science Society | | https://escholarship.org/uc/item/64x320w7 |  |
| Potthast, M., Kiesel, J., Reinartz, K., Bevendorff, J., Stein, B. | A Stylometric Inquiry into Hyperpartisan and Fake News | 2017 | ACL 2018 | | https://www.semanticscholar.org/paper/A-Stylometric-Inquiry-into-Hyperpartisan-and-Fake-Potthast-Kiesel/ed31e1225f6a76b469dfe4d022b235dc70be4390 |  |
| Fang, A., Ounis, I., Habel, P., Macdonald, C., Limsopatham, N. | Topic-centric Classification of Twitter User’s Political Orientation | 2015 | SIGIR '15: Proceedings of the 38th International ACM SIGIR Conference on Research and Development in Information Retrieval | | https://dl.acm.org/doi/pdf/10.1145/2766462.2767833 |  |

**Table S1: List of studies included, with their main features. Data valid at the time of data collection, 29.06.2021.**

## 1.3 The characteristics of the publications to be identified

For each study, we identified (if the question was applicable):

-year of publication

-authors, their affiliation

-title

-journal/publisher

-research questions

-country^^[[1]](#footnote-1)^^ and time period covered

-whether and how dynamics of polarization was examined

-which text analytics methods have been used (supervised or unsupervised etc.)

-if classification has been used: what the outcome is / what features have been used / how the training data have been annotated

-role of domain knowledge, whether the authors have tried to interpret it

-type of corpus (Twitter, etc.)

-restrictions of the corpus (keyword-search etc)

- conceptualization of polarization (eg. positional, affective)

- operationalization of polarization

-which layers of the political public sphere have been examined (politicians, the media, expert, or the lay public); if more than one layer: whether and how their relationship has been examined

-whether qualitative text analysis has been used

-important methodological novelty

## 1.4 Detailed descriptive statistics

| *Country of paper’s focus* | | *Affiliation country* | |
| --- | --- | --- | --- |
| USA | 91 | USA | 81 |
| United Kingdom | 9 | Italy | 14 |
| Italy | 6 | United Kingdom | 13 |
| Spain | 6 | Germany | 11 |
| Canada | 5 | Qatar | 10 |
| Germany | 5 | Canada | 8 |
| India | 3 | Spain | 7 |
| Scotland | 3 | India | 6 |
| Turkey | 3 | Ireland | 5 |
| Ukraine | 3 | France | 4 |
| Belgium | 2 | Israel | 4 |
| Chile | 2 | Switzerland | 4 |
| France | 2 | Bulgaria | 3 |
| Israel | 2 | Russian Federation | 3 |
| Japan | 2 | Turkey | 3 |
| Norway | 2 | Netherlands | 2 |
| Russia | 2 | Japan | 2 |
| Austria | 1 | Austria | 2 |
| Bolivia | 1 | Belgium | 2 |
| Catalonia | 1 | Chile | 2 |
| Colombia | 1 | Norway | 2 |
| Ecuador | 1 | Morocco | 1 |
| Indonesia | 1 | Sweden | 1 |
| Ireland | 1 | Denmark | 1 |
| Northern Ireland | 1 | Hungary | 1 |
| Palestine | 1 | Philippines | 1 |
| Philippines | 1 | Singapore | 1 |
| Poland | 1 | South Africa | 1 |
| Romania | 1 | Indonesia | 1 |
| South Africa | 1 | Romania | 1 |
| Non country-specific | 8 | Poland | 1 |
|  |  | Hong Kong | 1 |

**Table S2** Countries of the paper’s focus, and countries the authors are affiliated with (with frequency of occurrence). Country names follows the designation in the study.

| *Type of data source* | |
| --- | --- |
| Twitter | 66 |
| Congressional/Parliament speeches | 32 |
| news sites | 22 |
| speeches outside Parliament* | 8 |
| written public political documents** | 7 |
| Facebook | 6 |
| Reddit | 5 |
| blogs | 4 |
| texts produced by non-political experts*** | 4 |
| newspapers | 3 |
| Presidential candidates’ debates | 2 |
| texts produced by non-political organizations | 2 |
| wikis (Conservapedia, RationalWiki ) | 2 |
| YouTube comments | 2 |
| Online debate forums (4Forums.com,CreateDebate.com) | 1 |
| YouTube comments | 1 |
| books/magazine articles | 1 |
| Google Ngram | 1 |
| Google Search snippets | 1 |
| interview transcripts | 1 |
| political experts (political thinkers, commentators) | 1 |
| poll | 1 |
| Russian social network “VKontakte” | 1 |
| television transcripts (news show) | 1 |
| Tumblr | 1 |
| WhatsApp | 1 |

**Table S3** Type of data source with frequency of occurrence (*: presidential/presidential candidates' speeches, Campaign speeches, public statements, presidential candidacy announcements, **: party manifestos, candidate manifetos, party platforms, press releases, coalition agreements, ***: judges’ written opinions, business reports, scientific papers)

| *International collaborations* | | | *Multiple-authored publications without international collaboration* | | | *Single-authored publications* | |
| --- | --- | --- | --- | --- | --- | --- | --- |
| CHE | FRA | 1 | AUT | AUT | 1 | BEL | 1 |
| CHE | ITA | 1 | BEL | BEL | 1 | FRA | 1 |
| DEU | CHE | 1 | CAN | CAN | 6 | GBR | 1 |
| DEU | IRL | 1 | CHE | CHE | 1 | NOR | 1 |
| DEU | NLD | 1 | CHL | CHL | 1 | POL | 1 |
| DNK | AUT | 1 | DEU | DEU | 5 | QAT | 1 |
| GBR | DEU | 1 | ESP | ESP | 6 | SWE | 1 |
| GBR | IRL | 2 | FRA | FRA | 1 | USA | 7 |
| GBR | ITA | 2 | GBR | GBR | 4 |  |  |
| GBR | NLD | 1 | HUN | HUN | 1 |  |  |
| HKG | JPN | 1 | IDN | IDN | 1 |  |  |
| IND | GBR | 1 | IND | IND | 4 |  |  |
| IND | NLD | 1 | IRL | IRL | 3 |  |  |
| ISR | GBR | 1 | ISR | ISR | 1 |  |  |
| ITA | AUT | 1 | ITA | ITA | 9 |  |  |
| ITA | DNK | 1 | JPN | JPN | 1 |  |  |
| ITA | ESP | 1 | NOR | NOR | 1 |  |  |
| MAR | FRA | 1 | PHL | PHL | 1 |  |  |
| QAT | BGR | 3 | QAT | QAT | 1 |  |  |
| QAT | DEU | 2 | ROU | ROU | 1 |  |  |
| RUS | DEU | 1 | USA | USA | 57 |  |  |
| TUR | QAT | 2 | ZAF | ZAF | 1 |  |  |
| USA | BGR | 1 |  |  |  |  |  |
| USA | CAN | 2 |  |  |  |  |  |
| USA | CHL | 1 |  |  |  |  |  |
| USA | GBR | 4 |  |  |  |  |  |
| USA | IND | 2 |  |  |  |  |  |
| USA | IRL | 1 |  |  |  |  |  |
| USA | ISR | 3 |  |  |  |  |  |
| USA | QAT | 2 |  |  |  |  |  |
| USA | RUS | 2 |  |  |  |  |  |
| USA | SGP | 1 |  |  |  |  |  |
| USA | TUR | 1 |  |  |  |  |  |

**Table S4** Co-authorship of countries

| *Interdisciplinary collaborations* | | | *Multiple-authored publications without interdisciplinary collaboration* | | | *Single-authored publications* | |
| --- | --- | --- | --- | --- | --- | --- | --- |
| Social Sciences | Technology | 47 | Social Sciences | Social Sciences | 28 | Social Sciences | 13 |
| Social Sciences | Physical sciences | 3 | Technology | Technology | 61 | Technology | 1 |
| Social Sciences | Life Sciences & Biomedicine | 2 |  |  |  |  |  |
| Social Sciences | Arts & Humanities | 2 |  |  |  |  |  |
| Technology | Physical sciences | 3 |  |  |  |  |  |
| Technology | Life Sciences & Biomedicine | 3 |  |  |  |  |  |
| Technology | Arts & Humanities | 2 |  |  |  |  |  |
| Physical sciences | Life Sciences & Biomedicine | 2 |  |  |  |  |  |

**Table S5** Interdisciplinary collaborations. Web of Science classification^[[2]](#footnote-2)^ was used to categorize research areas into five broad categories, with one exception: Linguistics was not classified into Social Sciences, but into Technology. The reason for this was that we were primarily interested in whether authors work in interdisciplinary teams that include subject matter scientists with domain knowledge (sociologists, political scientists etc.) in addition to data analysts (computer scientists, computational linguists) etc.

## 1.5 Short introduction to the text analytic methods most used in the studies

There are standard political science approaches to define the political position of a text. These are scaling methods, i.e. they position the text along a numerical dimension. The **Wordscores** model was developed by Laver et al. (2003): it compares texts to other texts with a priori known positions by comparing the word frequencies in a given text with reference texts that define the extreme ends of the scale; in this sense it is a supervised method. **Wordfish** was developed by Slapin and Proksch (2008), and contrary to Wordscores, it is unsupervised as it does not require a priori positioned texts: it estimates an underlying latent variable reflecting ideology, based on word frequencies. The **Wordshoal** model (Lauderdale and Herzog, 2016) is an extension of Wordfish: while the latter recovers only one dimension, the former allows for the inclusion of further dimension(s) interfering with ideology. Wordshoal also allows the model to control for metadata, for example, the subject of the debate, because the nature of a word use variation can be different in different debates.

The following are classic NLP-methods. (As further reading on these methods we recommend Evans and Aceves’s paper (2016), and Ignatow and Mihalcea’s book (2017).)

The goal of **topic modeling** is to find latent topics in the corpus. This model assumes that there is a finite number of topics, which generate the documents. The topics are distributions over the words in the vocabulary. There are several different topic modeling techniques, which differ in their statistical assumptions, e.g. the structural topic model (STM), which is able to use document-level metadata that are allowed to affect topical prevalence or topical content (Wesslen 2018).

**Word embedding** is a representation of words based on their meaning, identifying meaning with the contexts of the word. Technically, words are embedded in a multi-dimensional semantic vector space, where words are positioned by their meanings, which is defined by their narrow textual environment. Two words will be positioned close or far from each other according to the similarity of their environment in the corpus. Word embeddings can be obtained by different language models and algorithms. There are generalizations of the method that give vector representations to larger text units (sentences, documents).

The above methods are **unsupervised** by default, i.e. they don't require preliminary assumptions or external knowledge, and the model learns the structure of the data itself. On the contrary, **supervised** methods require a pre-labelled dataset (for example the labels might be "liberal" and "conservative"). The aim of supervised learning models is to learn how to assign these labels to the texts. By dividing the labelled data into a training set and a test set, we can measure the model's performance.

Methods of **sentiment analysis** seek to assign a subjective state (an emotion or attitude toward an object) to the texts. Sentiment analysis can be performed by implementing both unsupervised and supervised approaches, where unsupervised approaches may for example use a dictionary of words pertaining to different states (e.g. negative / positive). One of the most elaborate dictionaries is LIWC (Linguistic Word County and Inquiry, Pennebaker et al. 2015). LIWC identifies words in a text belonging to predefined categories like basic emotions.

## 1.6 References

Arksey, H. & O’Malley, L. 2005. Scoping studies: towards a methodological framework. *International Journal of Social Research Methodology* 8 (1): 19–32. https://doi.org/10.1080/1364557032000119616

Evans, J. A., & Aceves, P. (2016). Machine Translation: Mining Text for Social Theory. *Annual Review of Sociology*, 42(1), 21–50. doi:10.1146/annurev-soc-081715-074206

Ignatow, G., & Mihalcea, R. F. (2017). *An Introduction to Text Mining: Research Design, Data Collection, and Analysis* (1 edition.). Los Angeles: SAGE Publications, Inc.

Lauderdale, B.E. & Herzog, A. 2016. Measuring political positions from legislative speech. *Political Analysis* 24 (3): 374–394. https://doi.org/10.1093/pan/mpw017

Laver, M., Benoit, K. & Garry, J. 2003. Extracting policy positions from political texts using words as data. *American Political Science Review* 97 (2): 311-331. <https://doi.org/10.1017/s0003055403000698>

Pennebaker, J.W., Booth, R.J., Boyd, R.L. & Francis, M.E. 2015. *Linguistic Inquiry and Word Count: LIWC2015*. Austin, TX: Pennebaker Conglomerates (www.LIWC.net).

Slapin, J.B. & Proksch, S.-O. 2008. A scaling model for estimating time-series party positions from texts. *American Journal of Political Science* 52 (3): 705–722. https://doi.org/10.1111/j.1540-5907.2008.00338.x

Wesslen, R. 2018. Computer-assisted text analysis for social science: Topic models and beyond. arXiv preprint on arXiv:1803.11045. https://doi.org/10.48550/arXiv.1803.11045

1. “Country of paper’s focus” was not always applicable, for example when a global social media site was involved in examining non-geographically delimited contributors to a particular debate. If a study examined more than one country, all of them were indicated separately in the table. [↑](#footnote-ref-1)
2. https://images.webofknowledge.com/images/help/WOS/hp_research_areas_easca.html [↑](#footnote-ref-2)
